# Phosphoprotein Phosphatase 1 but Not 2A Activity Modulates Coupled-Clock Mechanisms to Impact on Intrinsic Automaticity of Sinoatrial Nodal Pacemaker Cells

**DOI:** 10.3390/cells10113106

**Published:** 2021-11-10

**Authors:** Syevda Tagirova Sirenko, Ihor Zahanich, Yue Li, Yevgeniya O. Lukyanenko, Alexey E. Lyashkov, Bruce D. Ziman, Kirill V. Tarasov, Antoine Younes, Daniel R. Riordon, Yelena S. Tarasova, Dongmei Yang, Tatiana M. Vinogradova, Victor A. Maltsev, Edward G. Lakatta

**Affiliations:** Laboratory of Cardiovascular Science, National Institute on Aging, NIH, Baltimore, MD 21224, USA; syevda.tagirova@nih.gov (S.T.S.); zahanich@gmail.com (I.Z.); yueli@som.umaryland.edu (Y.L.); lukyanenkoy@nia.nih.gov (Y.O.L.); alexey.lyashkov@nih.gov (A.E.L.); zimanb@grc.nia.nih.gov (B.D.Z.); tarasovkv@mail.nih.gov (K.V.T.); antoine.younes@wanadoo.fr (A.Y.); riordond@grc.nia.nih.gov (D.R.R.); tarasovay@grc.nia.nih.gov (Y.S.T.); dongmei.brochet@nih.gov (D.Y.); vinogradovat@grc.nia.nih.gov (T.M.V.); maltsevvi@mail.nih.gov (V.A.M.)

**Keywords:** sinoatrial node cells, local Ca^2+^ releases, phosphoprotein phosphatase, endogenous phosphatase inhibitors, calyculin A, okadaic acid, phospholamban, ryanodine receptors, L-type Ca^2+^ channels, numerical model

## Abstract

Spontaneous AP (action potential) firing of sinoatrial nodal cells (SANC) is critically dependent on protein kinase A (PKA) and Ca^2+^/calmodulin-dependent protein kinase II (CaMKII)-dependent protein phosphorylation, which are required for the generation of spontaneous, diastolic local Ca^2+^ releases (LCRs). Although phosphoprotein phosphatases (PP) regulate protein phosphorylation, the expression level of PPs and phosphatase inhibitors in SANC and the impact of phosphatase inhibition on the spontaneous LCRs and other players of the oscillatory coupled-clock system is unknown. Here, we show that rabbit SANC express both PP1, PP2A, and endogenous PP inhibitors I-1 (PPI-1), dopamine and cyclic adenosine 3′,5′-monophosphate (cAMP)-regulated phosphoprotein (DARPP-32), kinase C-enhanced PP1 inhibitor (KEPI). Application of Calyculin A, (CyA), a PPs inhibitor, to intact, freshly isolated single SANC: (1) significantly increased phospholamban (PLB) phosphorylation (by 2–3-fold) at both CaMKII-dependent Thr^17^ and PKA-dependent Ser^16^ sites, in a time and concentration dependent manner; (2) increased ryanodine receptor (RyR) phosphorylation at the Ser^2809^ site; (3) substantially increased sarcoplasmic reticulum (SR) Ca^2+^ load; (4) augmented L-type Ca^2+^ current amplitude; (5) augmented LCR’s characteristics and decreased LCR period in intact and permeabilized SANC, and (6) increased the spontaneous basal AP firing rate. In contrast, the selective PP2A inhibitor okadaic acid (100 nmol/L) had no significant effect on spontaneous AP firing, LCR parameters, or PLB phosphorylation. Application of purified PP1 to permeabilized SANC suppressed LCR, whereas purified PP2A had no effect on LCR characteristics. Our numerical model simulations demonstrated that PP inhibition increases AP firing rate via a coupled-clock mechanism, including respective increases in the SR Ca^2+^ pumping rate, L-type Ca^2+^ current, and Na^+^/Ca^2+^-exchanger current. Thus, PP1 and its endogenous inhibitors modulate the basal spontaneous firing rate of cardiac pacemaker cells by suppressing SR Ca^2+^ cycling protein phosphorylation, the SR Ca^2+^ load and LCRs, and L-type Ca^2+^ current.

## 1. Introduction

**Figure 1:**
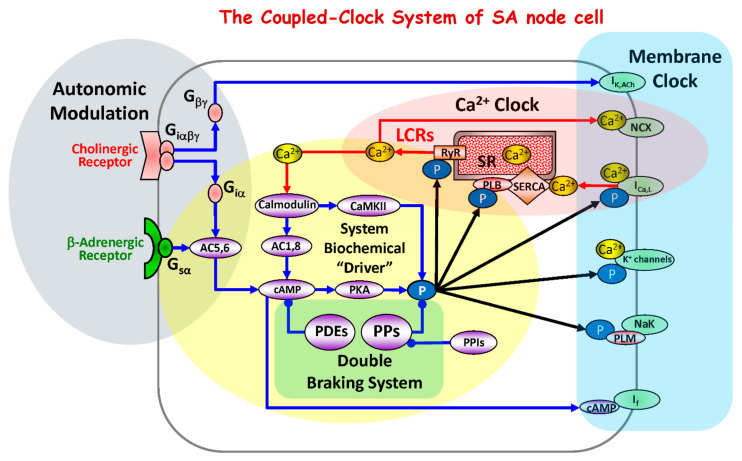
Schematic diagram of the coupled-clock system that includes four major sub-systems or functional modules: the sarcoplasmic reticulum, the Ca^2+^ clock; sarcolemmal ion channels and transporters, the membrane clock; biochemical drivers; and the autonomic modulation of the system components. The Ca^2+^ clock cycles Ca^2+^ via SR Ca^2+^ pump (SERCA) and Ca^2+^ release channels (RyRs). The membrane clock generates APs and interacts with the Ca^2+^ clock via multiple Ca^2+^-dependent mechanism, including NCX current that accelerates the diastolic depolarization. The system’s biochemical driver is cAMP, which is generated by Ca^2+^-activated AC1and AC8 and leads to activation of PKA. PKA and CaMKII increase phosphorylation of clock proteins (black arrows). The autonomic nervous system modulates the clock system via G protein-coupled receptor signaling. The cAMP level is kept in check by PDEs. The focus of the present study is to determine whether PPs, by keeping clock protein phosphorylation levels in check, form the double braking system with PDEs. Abbreviations: I_K_,_Ach_, acetylcholine-activated K^+^ current; NCX, Na^+^/Ca^2+^ exchanger; I_CaL_, L-type Ca^2+^ current; K^+^ channels, potassium channels; I_f_, hyperpolarization-activated current; Ca^2+^, calcium ions; LCRs, local submembrane Ca^2+^ releases; RyR, ryanodine receptors; SR, sarcoplasmic reticulum; SERCA, SR Ca^2+^ ATPase; CaMKII, calcium-calmodulin-dependent protein kinase II; PLM, phospholemman; PLB, phospholamban; P, phosphorylation; PKA, protein kinase A; cAMP, cyclic adenosine 3′,5′-monophosphate; AC, adenylyl cyclase; G_gsα_, G-protein coupled receptors stimulatory alpha subunit; G_iα_, G-protein coupled receptors inhibitory alpha subunit; G_iαβγ_, G-protein coupled receptors inhibitory alpha, betta, gamma subunits; G_βγ_, G-protein coupled receptors beta, gamma subunits; PPs, phosphoprotein phosphatases; PPIs, phosphoprotein phosphatase inhibitors; PDEs, phosphodiesterases.

The spontaneous action potential (AP) firing rate of sinoatrial nodal cells (SANC) is critically dependent on protein kinase A (PKA) and Ca^2+^/calmodulin-dependent protein kinase II (CaMKII)-dependent phosphorylation [1,2,3,4] (Figure 1). SR-generated, subsarcolemmal, rhythmic, local Ca^2+^ releases (LCRs) via RyR activate an inward Na^+^/Ca^2+^ exchange current (I_NCX_), accelerating the rate of diastolic depolarization and leading to an increase in the spontaneous AP firing rate (Figure 1). Basal state phosphorylation of SR Ca^2+^ cycling proteins and L-type Ca^2+^ channels by PKA and CaMKII in SANC is markedly higher than in ventricular myocytes (VM) and both (CaMKII and PKA) are required for the generation of basal rhythmic LCRs and spontaneous AP firing of SANC [5].

The signaling cascade that activates basal protein kinase activity in SANC is “feed-forward”, i.e., Ca^2+^ release stimulates CaMKII and PKA (via Ca^2+^-calmodulin activated adenylyl cyclases (ACs)) and these kinases, by phosphorylating membrane clock proteins and SR Ca^2+^ cycling proteins, increase SR Ca^2+^ release, which further activates Ca^2+^-calmodulin activated ACs [6,7] and CaMKII (Figure 1).

The high basal level of phosphorylation in SANC is partly due to the increased level of cAMP, which is substantially higher in SANC than in ventricular myocytes [1,3,7]. Constitutive activity (in the absence of autonomic receptor activation) of Ca^2+^-dependent ACs supports both the increased level of cAMP and high protein phosphorylation in the basal state [3,7].

Potent restraining mechanisms of this feed-forward signaling axis are required to maintain the basal SANC spontaneous AP firing rate near its mid-range: an increase or reduction in phosphorylation from the basal level by β-adrenergic or cholinergic receptor stimulation, induces respective increases or decreases in AP firing rate [1,3,8,9]. The constitutive activation of ACs in SANC is counterbalanced by constitutive activation of phosphodiesterases (PDEs) (Figure 1), which are enzymes that degrade cAMP and regulate cyclic nucleotide signaling. SANC have a high basal level of PDE activity. For example, a broad-spectrum PDE inhibitor IBMX produces a ~nine-fold increase in the cAMP level, signifying that PDEs provide negative feedback regulation to limit and fine-tune the basal cAMP level [5,10,11]. High basal PDE activity controls not only basal cAMP level but also spatial cAMP distribution within the cell. Rapid degradation of cAMP by PDEs provides functional barriers to cAMP diffusion, and therefore creates discrete intracellular pools of cAMP, functionally and spatially compartmentalizing signaling pathways within the cell [12,13].

An increase in phosphoprotein phosphatase activity is also a potential “brake” on the feed-forward basal phosphorylation-dependent signaling that regulates SANC AP firing (Figure 1). In VM, two major protein phosphatases, PP1 and PP2A, are responsible for 90% of protein dephosphorylation in the heart [14]. Although they often target the same proteins via Ser^16^/Thr^17^ sites, their relative contributions to specific protein substrates is different [15]. PP2A primarily dephosphorylates L-type Ca^2+^ channels and PP1 primarily dephosphorylates phospholamban (PLB). We have previously demonstrated that basal phosphatase activity in VM regulates protein kinase-dependent phosphorylation of SR Ca^2+^ cycling proteins, preventing VM sparks to become spontaneous rhythmic LCRs as in SANC [16]. The application of an exogenous phosphoprotein phosphatases (PP) inhibitor, Calyculin A (CyA) [17,18], to rabbit SANC increased the AP firing rate, cyclic AMP level, ATP production, and the AP-induced Ca^2+^ transient amplitude [18]. In both rabbit SANC and canine Purkinje fibers, CyA also increased I_f_ current amplitude [19,20].

However, neither the expression properties and protein levels of specific phosphatases and endogenous phosphatase inhibitors nor the impact of basal phosphatase activity on Ca^2+^ cycling protein phosphorylation on spontaneous LCRs and other players of the oscillatory coupled-clock system have been studied.

Here, we determined the abundance of RNA transcripts and level of protein expression of PP1 and PP2A in lysates of isolated rabbit SANC. Considering that the catalytic site of PP1 is extremely sensitive to several endogenous inhibitors [21], we also measured the abundance of mRNA transcripts and protein levels of I-1, KEPI and DARPP-32. We also assessed these transcripts in left ventricle cells (LVC) for comparison with their expression abundance in SANC. In intact, freshly isolated SANC, we determined the effects of pharmacological inhibition on phosphorylation of Ca^2+^ cycling proteins (i.e., PLB) as well as L-type Ca^2+^ current amplitude, AP parameters, AP-triggered Ca^2+^ transients, and spontaneous AP firing rates. We also assessed the effects of these inhibitors on spontaneous diastolic LCRs in intact SANC and assessed the effects of purified PP1 and PP2A on LCR characteristics in permeabilized SANC. Finally, we determined whether a numerical model [22], fine-tuned by experimentally observed biophysical effects produced by PP inhibition in SANC, could quantitively predict changes in AP firing rate in response to the PP inhibition via a coupled-clock mechanism.

## 2. Materials and Methods

The study was performed in accordance with the Guide for the Care and Use of Laboratory Animals published by the National Institutes of Health (NIH Publication number 85–23, revised 1996). The experimental protocols were approved by the Animal Care and Use Committee of the National Institutes of Health (protocol#457-LCS-2024). Detailed descriptions of methods are presented in the Online Data Supplement.

### 2.1. Isolation of Cardiac Cells

Single, spontaneously beating SANC and left ventricular cardiomyocytes (LVC) were isolated from the hearts of New Zealand rabbits (Charles River Laboratories, Wilmington, MA, USA) by enzymatic digestion as described previously [11,23].

### 2.2. RNA Extraction, cDNA Synthesis, RT-QPCR

RNA was extracted from isolated rabbit left ventricular (LV), or SANC with RNeasy Mini Kit (Qiagen, Germantown, MD, USA) with DNAse on column digestion according to manufacturer protocol. A total of 2 μg of total RNA was used for cDNA preparation in 50 μL reaction volume with MMLV reverse transcriptase (Life Technologies, Foster City, CA, USA) using manufacturer recommended conditions with random hexamers for priming. For each synthesis of cDNA, we used no template control for the detection of possible contamination and no RT control for tracing of possible genomic DNA presence.

### 2.3. Western Blotting

For the detection of protein phosphatases and endogenous protein phosphatase inhibitors, cells isolated from rabbit LV and sinoatrial nodes were lysed, transferred to membranes, incubated with antibodies against PPP1CA (Sigma-Aldrich, St. Louis, MO, USA SAB5300221, 1:2500), PPP2CA (Millipore, Bedford, MA, USA 06-222, 1:1000), Inhibitor 1 (Abcam, Cambridge, MA, USA ab40877, 1:10000), KEPI (Biorbyt, Cambridge, UK orb128558, 1:2000), DARPP-32 (Santa Cruz Biotechnology, Dallas, TX, USA sc-271111, 1:1000), or Sarcomeric Alpha-Actinin (Sigma-Aldrich, St. Louis, MO, USA A7811, 1:5000) and developed with Pierce SuperSignal West Pico or West Dura ECL substrate kits (ThermoFisher Scientific, Waltham, MA, USA).

The detection of site specific PLB phosphorylation sites was performed in SANC as previously described [24].

### 2.4. Spontaneous APs and Ca^2+^ Current Recordings in Intact SANC

A perforated patch-clamp technique was employed to record APs and a ruptured patch-clamp technique was used to record Ca^2+^ current using an Axopatch-200B patch-clamp amplifier (Axon Instruments, Foster City, CA, USA) at 35 ± 0.5 °C. Only regularly spontaneously beating SANC were chosen for recordings of either APs or currents as described previously [4,25].

### 2.5. Confocal Imaging of AP-Induced Ca^2+^ Transient and Local Subsarcolemmal Ca^2+^ Releases (LCR) in Intact SANC

AP-induced global Ca^2+^ transients and spontaneous LCRs were measured in SANC and loaded with a Ca^2+^ indicator, Fluo-4AM (Thermo Scientific, Waltham, MA, USA) (0.01 mM, 15 min) 35 ± 1 °C, via a confocal microscope (Zeiss LSM510, Jena, Germany) in the line-scan mode as described previously [26].

### 2.6. SR Ca^2+^ Content and LCRsin Permeabilized SANC

A subset of SANC was permeabilized with 0.01% saponin. After saponin washout, the solution was changed to the recording solution with the addition of 0.03 mM fluo-4 pentapotassium salt (Thermo Scientific, Waltham, MA, USA) and 150 nmol/L free [Ca^2+^]_i_. SR Ca^2+^ content and LCRs were measured with a confocal microscope (Zeiss LSM510, Jena, Germany) in the line-scan mode at 35 ± 0.5 °C, as previously described [27].

### 2.7. Immunolabeling of RyR2 in SANC

Immunolabeling of RyR2 total and phosphorylated RyR2 at Ser^2809^ were performed in SANC as previously described [28]. Dual confocal images of central sections of SANC were obtained with lasers 633 nm and 543 nm via a Zeiss LSM 510 (Carl Zeiss Inc., Jena, Germany).

### 2.8. Numerical Modeling

Numerical model simulations employed a model of rabbit SANC that portrays the pacemaker cell function as a coupled system of a Ca^2+^-clock, regulated by intracellular Ca^2+^ cycling and a surface membrane or “M clock” regulated by the ensemble of surface membrane electrogenic molecules [22].

### 2.9. Statistics

Data are presented as mean ± SEM. Statistical significance of differences between means was evaluated by Student’s *t*-test or analysis of variance (ANOVA) when appropriate. A value of *p* < 0.05 was considered statistically significant.

## 3. Results

### 3.1. Protein Phosphatases and Protein Phosphatase-1 Inhibitor Transcript Abundance and Protein Levels in SANC, RAC and LVC

**Figure 2:**
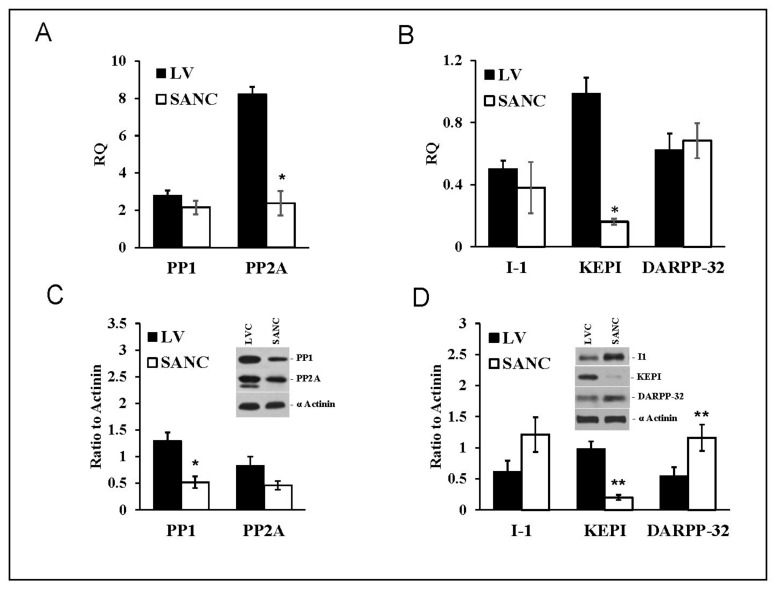
mRNA expression (**A**,**B**) and protein abundance (**C**,**D**) (Mean+/−SEM) patterns of PP1, PP2A, and PP1 endogenous inhibitors (I-1, KEPI and DARPP-32) normalized to β-tubulin (mRNA) or α-actinin in LVC and SANC (*n* = 6 independent hearts analyzed per group); * *p* < 0.05, ** *p* < 0.01 compared to LVC. For alternative transcript names see Table S1. For Primers and probes used for RT-QPCR see Table S2.

SANC showed similar profiles of PP1 and PP2A transcript abundance and protein expression (Figure 2A). PP1 transcripts in LVC were equivalent to those in SANC, but PP2A transcript abundance in LVC exceeded that in SANC four-fold (Figure 2A). Whereas I-1 and DARPP-32 transcript abundances were similar in all cell types (Figure 2B), KEPI transcript abundances in LVC exceeded those in SANC (Figure 2B).

PP1 protein levels in LVC exceeded those in SANC; there was also a non-significant trend for LVC PP2A levels to be higher than those in SANC (Figure 2C). The endogenous PP inhibitor I-1(PPI-1) protein levels in SANC tended to be higher than that in LVC (Figure 2D). DARPP-32 levels in SANC also significantly exceeded that in LVC (Figure 2D); in contrast, KEPI protein was markedly reduced in SANC compared to LVC. Thus, compared to LVC, PP1 levels were lower in SANC, while those of endogenous PP1 inhibitors (DARPP-32 and I-1) were higher, a pattern that might be expected, because protein phosphorylation status in SANC is increased compared to that in LVC [27].

### 3.2. Effect of PP Inhibition on Target Protein Phosphorylation in SANC

**Figure 3:**
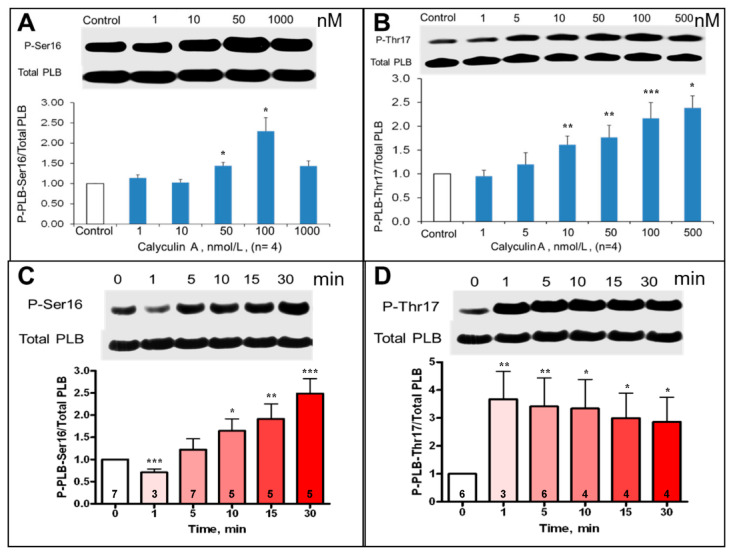
Average effects of CyA on the magnitude of SANC PLB phosphorylation at PKA-dependent Ser^16^ and CaMKII-dependent Thr^17^ sites. (**A**,**B**) Concentration dependence (30 min CyA treatment, *n* = 4). (**C**,**D**) Rates of phosphorylation in response to 100 nmol/L CyA. Representative Western blots of phosphorylated PLB and total PLB are presented in the upper part of each panel. Number of samples is shown in each column. * *p* < 0.05, ** *p* < 0.01, *** *p* < 0.001 vs. control.

PLB phosphorylation at PKA-dependent and CaMKII-dependent sites in intact, isolated SANC was assessed prior to and during inhibition of PP by CyA over 1–1000 nmol/L range (Figure 3A,B).

CyA induced a significant, ~2.5–3-fold concentration-dependent increase in PLB phosphorylation at both PKA-dependent Ser^16^ and CaMKII-dependent Thr^17^ sites (Figure 3A,B). Interestingly, the increase in PLB phosphorylation at Thr^17^ in response to 100 nmol/L CyA was rapid and complete within one minute (Figure 3D), while PLB phosphorylation at Ser^16^ increased gradually and reached its peak over 30 min (Figure 3C). Inhibition of PP activity by CyA in intact SANC also significantly increased phosphorylation of RyR at Ser^2809^ sites (Figure S1), which were phosphorylated by both PKA and CaMKII [2].

### 3.3. Effects of PP Inhibition by CyA or Purified PP1 and PP2A on LCRs in Permeabilized SANC

**Figure 4:**
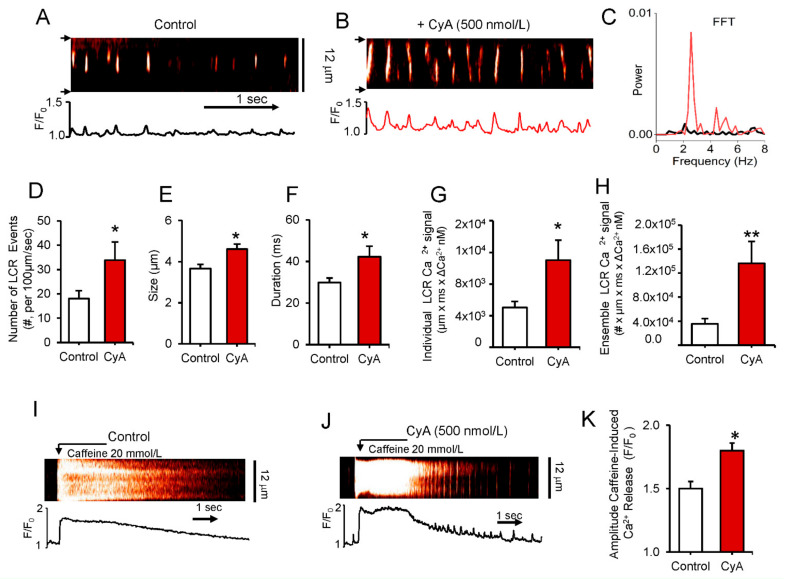
(**A**,**B**) Representative confocal line-scan images and (**C**) a fast Fourier transform (FFT) of Ca^2+^ oscillations in a permeabilized SANC bathed in 150 nmol/L [Ca^2+^] in control conditions and during superfusion with 500 nmol/L CyA. (**D**–**H**) Average changes in local Ca^2+^ release characteristics (LCRs) in control and during superfusion with 500 nmol/L CyA (*n* = 6): (**D**) Number of LCR events per 100 µm of the line-scan image and during a 1-sec time interval; (**E**) LCR size (as FWHM, the full width at half-maximum amplitude); (**F**) LCR duration (as FDHM, the full duration at half-maximum amplitude); (**G**) Ca^2+^ signals of Individual LCRs (µmxmsxΔCa^2+^ nmol/L); (**H**) Ca^2+^ signals of the LCR ensemble (the integrated Ca^2+^ signal of all LCRs), * *p* < 0.05, ** *p* < 0.01 paired *t*-test vs control, *n* = 6. Representative confocal images (**I**,**J**) and the average amplitude of the SR Ca^2+^ release (**K**) induced by a rapid spritz application of 20 mM caffeine in permeabilized SANC during 500 nmol/L CyA superfusion (*n* = 12) and in control (*n* = 7). * *p* < 0.05, 2-tailed *t*-test.

To determine the direct effects of PP on Ca^2+^ clock functions without interference of ion channels (e.g., L-type Ca^2+^ channels), we applied CyA to saponin-permeabilized SANC (Figure 4). CyA increased the peak of rhythmic LCR frequency as assessed by Fast Fourier transform analysis (FFT) and shifted it to a higher frequency from 2.10 Hz to 2.57 Hz in the example in Panel C and by 50% on average from 2.2 ± 0.2 to 3.2 ± 0.4 Hz (*n* = 4).

The average effects of CyA on LCR characteristics in permeabilized SANC are illustrated in Figure 4 (Panels D to H). CyA increases average LCR frequency by more than 80% (Panel D), average LCR size by 26% (Panel E) and LCR duration by 42% (Panel F). The mean Ca^2+^ signal of individual LCRs increases 2.2-fold (Panel G), and CyA increased the mean ensemble LCR Ca^2+^ signal about four-fold (Panel H). To determine whether an increase in the ensemble LCR Ca^2+^ signal is associated with an increase in the SR Ca^2+^ load in permeabilized SANC, we applied a pulse of caffeine, which rapidly empties the SR Ca^2+^ store before and after CyA application (Figure 4I,J). On average CyA induced a 20% increase in the mean amplitude of the caffeine-induced Ca^2+^ transients (Figure 4K). Thus, a 20% increase in the caffeine-induced SR Ca^2+^ release was accompanied by a four-fold increase in the ensemble of LCR Ca^2+^ signal, suggesting an increase in kinetics of SR Ca^2+^ cycling in response to PP1 inhibition by CyA. This idea is consistent with the effects of CyA on PLB and RyR phosphorylation (Figure 3 and Figure S1, respectively). In contrast to CyA, superfusion of permeabilized SANC with okadaic acid produced no changes in LCR characteristics (Figure S2).

**Figure 5:**
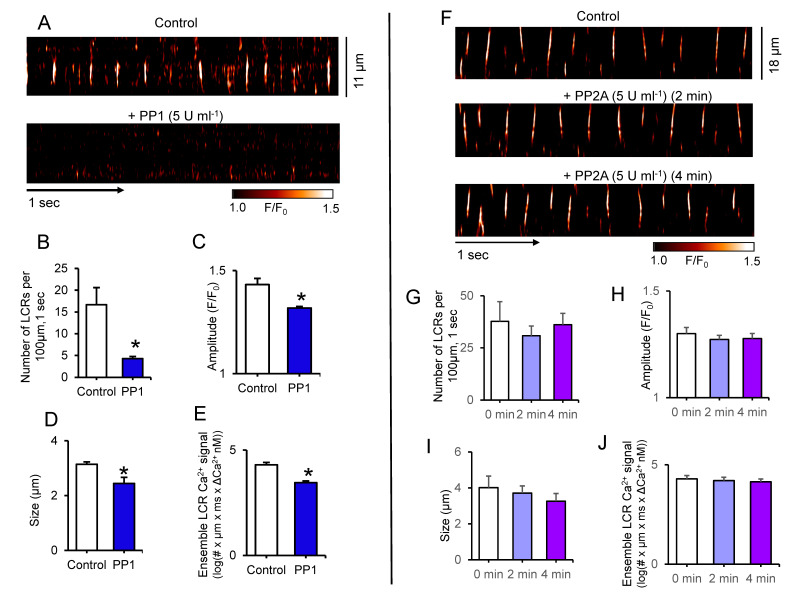
Representative confocal line-scan images of LCRs in permeabilized SANC bathed in 150 nmol/L [Ca^2+^] in control conditions and during incubation with purified PP1 (5 U mL^−1^) (**A**) or PP2A (5 U mL^−1^) catalytic subunits (**F**). Changes in local Ca^2+^ release (LCRs) characteristics before and during exposure to PP1 (*n* = 4) (**B**–**E**) or PP2A (*n* = 6) (**G**–**J**); (**B**,**G**) Number of LCR events per 100 µm of the line-scan image and during a 1-sec time interval; (**C**,**H**) LCR amplitude (F/F_0_); (**D**,**I**) LCR size (as FWHM, the full width at half-maximum amplitude); (**E**,**J**) Average ensemble of LCR Ca^2+^ signals (as log of the integrated Ca^2+^ signal produced by each LCR; #-represents number of LCR events). * *p* < 0.05, the paired *t*-test vs. control (**B**–**E**); one way ANOVA (**G**–**J**).

Figure 5 shows the effects of direct application of PP1 or PP2A enzymes to permeabilized SANC. While application of PP1 markedly suppressed LCR number, amplitude and size and decreased ensemble of LCR Ca^2+^ signal by ~7.4-fold, there were no changes in LCR characteristics in response to PP2A application (Figure 5). The lack of an effect of purified PP2A on LCRs in permeabilized SANC indicates that PP1 activity but not PP2 activity suppresses spontaneous SR Ca^2+^ cycling of SANC.

### 3.4. Effect of PP Inhibition on AP Characteristics and Spontaneous AP Firing Rate in Spontaneously Beating SANC

**Figure 6:**
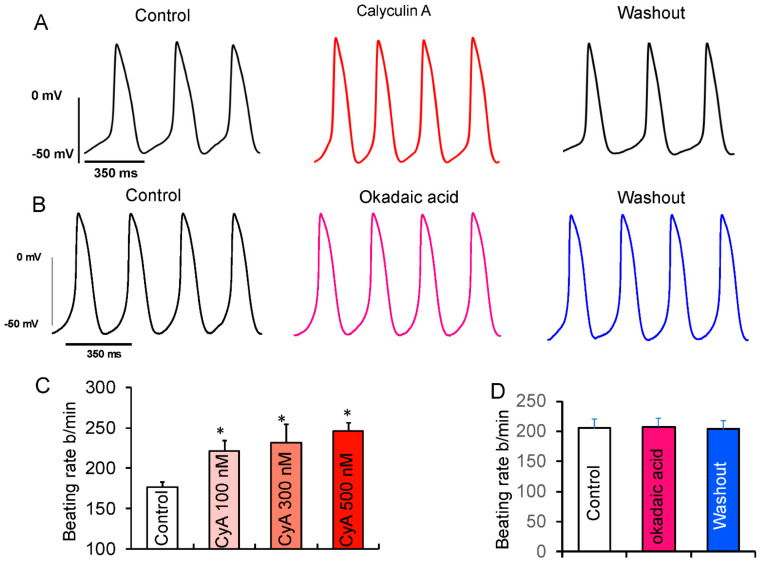
Effects of suppression of PP activity by CyA or OKA on spontaneous beating of isolated rabbit SANC. (**A**) Representative example of APs recorded in SANC before and during superfusion with 100 nmol/L CyA and following washout of CyA. (**B**) Representative example of SANC APs recorded prior to and during superfusion with 100 nmol/L OKA and after OKA washout. (**C**) The average increase in the spontaneous AP firing rate did not significantly differ in response to 100 (*n* = 10), 300 (*n* = 5) or 500 nmol/L (*n* = 6) of CyA. (**D**) Average spontaneous AP firing rate responses to OKA (*n* = 6). * *p* < 0.05 vs. control.

According to the coupled-clock theory of pacemaker cell function, the observed CyA-induced increase in LCR characteristics (Figure 4) and those effects of direct application of PP1 would be expected to increase the spontaneous basal AP firing rate. Previous studies demonstrated that CyA at both high (1 µM) [18] and low (100 nM) [17] concentrations increased SANC beating rate. We studied the effects of 100, 300, and 500 nM of CyA on the firing rate in rabbit SANC. We observed that CyA levels as low as 100 nM significantly increased the spontaneous AP firing rate in SANC by ~25% (Figure 6). The average effects of PP inhibition by CyA on AP parameters are listed in Table S3. The increase in the diastolic depolarization rate (DD slope) was accompanied by a decrease in the spontaneous SANC cycle length (SANC cycle), a decrease in the AP duration at 75% (APD75), a decrease in the time to the onset of non-linear diastolic depolarization (TNLDD, see methods), and an increase in the SANC firing rate at all CyA concentrations (100–500 nmol/L).

To distinguish whether these effects of CyA (Figure 6) were mediated via inhibition of PP1, PP2A, or both, we performed additional experiments employing a selective PP2A inhibitor okadaic acid (100 nmol/L). Okadaic acid also inhibits PP1, but with a 100-fold lower potency. It was shown that when applied to living cells at 100 nmol/L concentration, it does not inhibit PP1 [29]. Several studies have successfully used a 100 nmol/L concentration of okadaic acid to investigate the role of PP2A in VM [30,31].

In contrast to the effect of CyA, there was no increase in the spontaneous AP firing rate after inhibition of PP2A by okadaic acid (Figure 6B,D), suggesting that the observed effects of CyA on the basal AP firing rate were largely mediated by the inhibition of PP1, consistent with the lack of effects of purified PP2A on LCR characteristics (Figure 5).

### 3.5. Effect of PP Inhibition on Ca^2+^ Release during AP Firing Rate in Intact SANC

**Figure 7:**
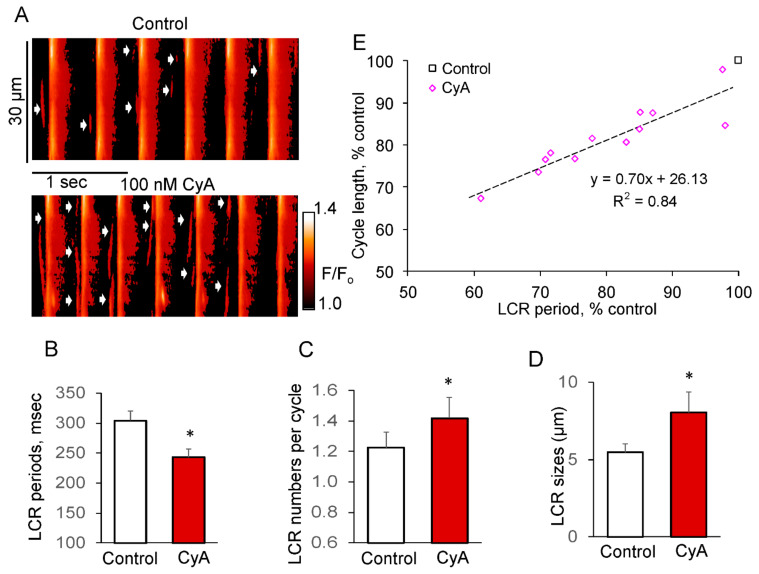
PP inhibition by CyA increases the LCR number and size of LCRs in SANC during spontaneous AP firing. (**A**) Confocal line-scan image of a representative SANC prior to and during exposure to 100 nmol/L CyA; CyA-induced changes in: (**B**) LCR period; (**C**) LCR number per cycle; (**D**) LCR size (*n* = 12). (**E**) PP inhibition-induced changes in the LCR periods predict the concurrent changes in the AP cycle lengths (*n* = 12). * *p* < 0.05 vs. control.

Figure 7 shows representative confocal line-scan images of a fluo-4 loaded spontaneous AP firing SANC prior to and during exposure to CyA. The increase in AP firing rate induced by 100 nmol/L CyA (Figure 7A) was accompanied by an acceleration in the decay of AP-induced Ca^2+^ transient indicated by the reduction in the duration of the Ca^2+^ transient amplitude at 50% (T_50_: from 104.6 ± 7.2 to 86.7 ± 5.4 ms, *p* < 0.05, *n* = 12 SANC) and at 90% (T_90_: from 206.2 ± 16.5 to 164.5 ± 11.1 ms, *p* < 0.003, *n* = 12 SANC). This effect is consistent with an increase in SR Ca^2+^ pumping rate that is expected to occur concurrently with CyA effects to increase PLB phosphorylation (Figure 3).

PP inhibition in spontaneously beating SANC significantly increased the LCR size by ~50% and number of LCRs per spontaneous cycle by ~20%, while reducing the LCR period by about 20%. The reduction in the LCR periods was accompanied by a decrease in the spontaneous SANC cycle lengths, and there was a close correlation between these parameters (R^2^ = 0.84, Figure 7E).

### 3.6. Effect of PP Inhibition by CyA on L-Type Ca^2+^ Current in Intact Voltage-Clamped SANC

**Figure 8:**
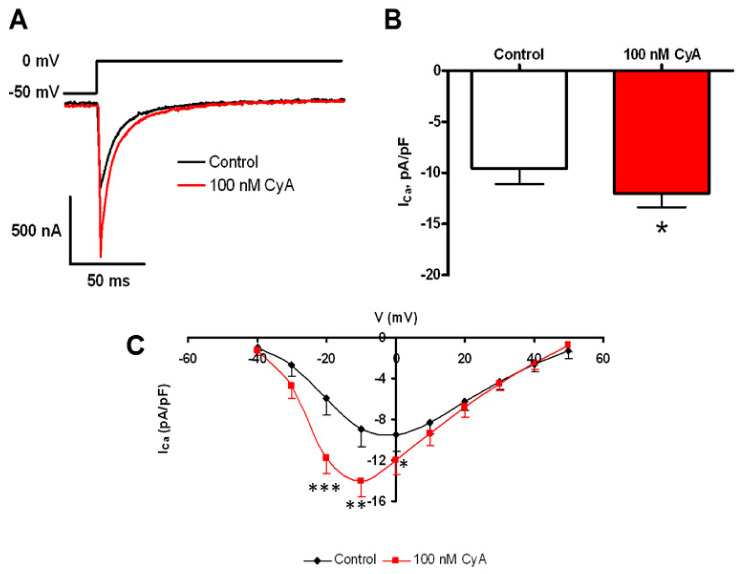
PP inhibition-induced potentiation of L-type Ca^2+^ current amplitude. (**A**) The voltage clamp protocol and representative original L-type Ca^2+^ current recordings prior to and during PP inhibition by CyA. Average effects of CyA on the amplitude (**B**) and current-voltage relationship (**C**) of I_Ca_ in SANC (*n* = 9). L-type Ca^2+^ current amplitudes were normalized to membrane capacitance. * *p* < 0.05, ** *p* < 0.01, *** *p* < 0.001.

Ca^2+^ influx via L-type Ca^2+^ current is a crucial component of Ca^2+^ cycling that sustains LCR activity in intact SANC [32], and L-type Ca^2+^ channels are likely targets of PP [30]. Figure 8 shows that PP inhibition by CyA augments the amplitude of I_CaL_ by about 30% and shifts its I–V relationship by ~10 mV leftward (*n* = 9). This result is consistent with a suppression of basal-state Ca^2+^ influx via L-type Ca^2+^ channels by basal phosphatase activity.

### 3.7. Numerical Modeling of the Effect of PP Inhibition by CyA in SANC

**Figure 9:**
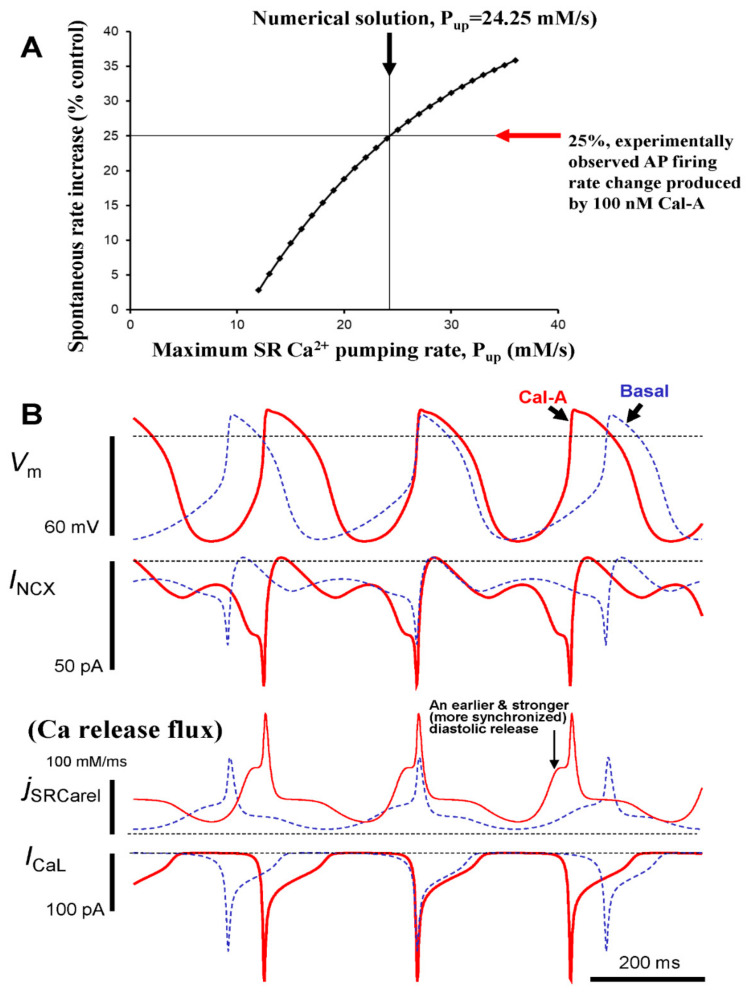
Numerical model simulations of the experimentally defined effects of protein phosphatase inhibition by CyA on spontaneous AP firing rate of rabbit SANC. (**A**) A numerical solution in which the maximum SR Ca^2+^ pumping rate, P_up_ was increased to 24.25 mM/s, reproduced the experimentally observed increase in AP firing rate by 25% in the presence of 100 nM of CyA. (**B**) Simulations of the membrane potential (V_m_), NCX current (I_NCX_), L-type Ca^2+^ current (I_CaL_), and Ca^2+^ release flux (j_SRCarel_) before (dashed line) and during CyA application (CyA, solid line).

We numerically simulated the biophysical effects of PP inhibition by 100 nM CyA in SANC (Figure 9). The quantitative impacts of increased protein phosphorylation on the diastolic LCRs’ release characteristics and spontaneous beating rate were explored by simultaneously increasing the maximum SR Ca^2+^ pumping rate (P_up_), mimicking the effect of PLB phosphorylation, and by increasing the maximum conductance of I_CaL_ (g_CaL_). 

In accordance with our experimental results, the purpose of our numerical modeling was to combine the effects of I_CaL_ and PLB phosphorylation in a biophysical detailed model to ascertain whether these effects can explain the PP firing rate change observed experimentally during PP inhibition. Because our experimental data showed that the maximum peak I_CaL_ is increased by 100 nmol/L CyA, on average from 9.6 to 14.2 pA/pF (Figure 8), g_CaL_ was increased in the model by 47.9% (Figure 9). Because PLB phosphorylation substantially increases (>2 fold vs. control, Figure 3), CyA surely increases P_up_. This magnitude of PLB phosphorylation increase is, indeed, very large, actually close to an extreme change, and comparable to that (also ~2.5 times) reported for the maximum effect of PDE inhibition by isobutylmethylxanthine (IBMX) in rabbit SANC [11]. The specific changes of P_up_ in intact SANC that result from increases in PLB phosphorylation, however, are unknown and cannot be assessed experimentally during spontaneous AP firing. Therefore, we explored model predictions when P_up_ was gradually increased whereas g_CaL_ was instantly increased, (by 47.9%, from 0.46 to 0.69 nS/pF, see above). This parameter sensitivity analysis allowed us to determine whether a numerical solution for the experimentally observed chronotropic effect of CyA to increase AP firing rate by 25% could be found with a reasonable P_up_ increase (i.e., within reported PLB modulatory capacity on P_up_). A numerical solution for the experimental effect of CyA increasing the AP firing rate by 25% was indeed identified in response to an approximate doubling of P_up_ in the model from 12 to 24.25 mM/s (Figure 9A). Of note, the CyA-induced increase in g_CaL_ alone (i.e., without a P_up_ increase) results only in a small change in the AP firing rate (2.8%), i.e., insufficient to account for the experimentally observed rate change of 25%.

To gain further insight into the biophysical mechanism of the AP firing rate increase caused by PP inhibition in response to CyA, the kinetics of key components of the system were numerically simulated (Figure 9B), including I_NCX_, I_CaL_, and diastolic SR Ca^2+^ flux. Model simulations predicted that PP inhibition resulted in earlier and stronger diastolic Ca^2+^ release flux, consistent with the experimental observation of a concurrent reduction in LCR period and increase in LCR amplitude (Figure 4 and Figure 7). The earlier and stronger diastolic Ca^2+^ release, in turn, activated, an earlier and stronger I_NCX_ (Figure 8B) that accelerated diastolic depolarization and the activation of I_CaL_.

Our immunolabeling results indicated that Ser2809 phosphorylation normalized to total RyR increased by 38.5% by CyA (from 1.3 to 1.8, Figure S1). Because this site is known to be phosphorylated by both PKA and CaMKII, it was uncertain how this increase in phosphorylated RyR translates into the Ca^2+^ release formulation in the model. To specifically address this issue, we performed an additional parametric sensitivity analysis to determine the robustness of our simulation results for CyA with respect to this increase in RyR phosphorylation. In the basal state model simulation, RyR opening rate (k_s_) was set to 250 μs^−1^. In our sensitivity analysis, k_s_ varied over a wide range, from 100 μs^−1^ (which is substantially below basal RyR opening rate) to 400 μs^−1^ (which is substantially above basal RyR opening rate) in increments of 10 μs^−1^ (total 31 models were tested). The model simulations showed that changes in the RyR opening rate over this broad range of ks applied on top of g_CaL_ and P_up_ increases (Figure 9, marked by arrows for 25% rate increase) did not substantially affect the 25% AP firing rate increase generated by g_CaL_ and P_up_ alone (Figure S3).

Because I_f_ was not measured experimentally in our study, we also performed a parametric sensitivity analysis of our numerical model to test whether our simulation of CyA effects was robust with respect to possible changes in I_f_. A classical study [33] showed that cAMP can shift I_f_ activation by up to 7.8 mV to more depolarized potential. Thus, we also shifted the respective model parameter V_If,1/2_ (the half maximum activation voltage of I_f_) by 7.8 mV, i.e., from −64 mV to −56.2 mV. Our simulations, however, revealed that the AP firing rate remained essentially unchanged (within 1%) when this increase in sensitivity to hyperpolarization of I_f_ was applied on top of I_CaL_ and P_up_ increases (Figure 9, marked by arrows for 25% rate increase). On the other hand, it was also suggested [19] that CyA modulates I_f_ in a different way, i.e., not by shifting V_If,1/2_ but by increasing the maximum I_f_ conductance by 39.6%. We also tested this mechanism by increasing the maximum I_f_ conductance (g_h_ parameter) in our model by 39.6% from 0.4 to 0.5584 nS/pF. This simulation also revealed only a minor increase (within 1%) in the AP firing rate. Thus, our simulations showed that neither the shift in V_If,1/2_ nor a major increase in I_f_ conductance influenced our result of a 25% rate increase by CyA application (Figure 9A).

## 4. Discussion

### 4.1. Basal PP1 Activity Modulates a Coupled-Clock System That Drives Spontaneous Automaticity of SANC

Basal protein phosphorylation in SANC is enabled by Ca^2+^-calmodulin activated adenylyl cyclases type 1 and 8, and CaMKII [2,3,4,7]. Phosphorylation of intracellular Ca^2+^ cycling proteins (Ca^2+^ clock) and surface membrane electrogenic proteins (membrane clock) at PKA and CAMKII-dependent sites in SANC are required for normal operation of a dynamic coupled-clock system that determines the spontaneous basal-state AP firing rate [3] and its AP in response to G protein coupled receptor stimulation [1]. The present results show that the effects of PP1 inhibition to increase AP firing rate result from integrated changes in functions of sarcolemmal (“M clock”) and intracellular (“Ca^2+^ clock”) proteins, including amplification of L-type Ca^2+^ current amplitude, leading to increases in Ca^2+^ influx and SR Ca^2+^ load, accompanied by changes in LCR characteristics. CyA-induced increase of the SANC AP firing rate in our study resulted from inhibition of PP1 because inhibition of PP2A by okadaic acid had no effect on the AP firing rate of intact SANC (Figure 6) or on LCR characteristics in permeabilized SANC (Figure S2). Further, exposure of permeabilized SANC to purified PP2A enzyme had no effect on LCRs, in contrast to a marked effect on LCR characteristics induced by PP1 enzyme in permeabilized SANC (Figure 5) or by CyA in intact SANC (Figure 7). The PP inhibition induced an increase in the kinetics of SR Ca^2+^ cycling, which resulted, in part at least, from increased PLB phosphorylation, (Figure 3) leading to an increased rate of Ca^2+^ pumping into SR, manifested in intact, spontaneously firing SANC as an increase in the kinetics of the decay rate of the AP-induced Ca^2+^ transient.

### 4.2. PP Transcript and Endogenous PP Inhibitor mRNA, and Protein Expression in SANC

It has been previously documented that basal phosphorylation levels of PLB and RyR are substantially lower in LVC than in SANC [3]. The present results (Figure 2) demonstrate that PP1 protein level and PP2A transcript abundance are both substantially higher in LVC than in SANC. This may explain, in part, why basal phosphorylation of Ca^2+^ cycling proteins in LVC is suppressed compared to that in SANC. PP1 is the major phosphatase that dephosphorylates PLB in LVC [34]. Immunolabeling of PP1 endogenous phosphatase inhibitors I-1 and DARPP-32 proteins were approximately two-fold greater in SANC than in LVC (Figure 2C,D), and KEPI was about four-fold lower in SANC than in LVC (Figure 2D). These cell type differences appear to be due to differences in protein degradation because mRNA transcripts for I-1, DARPP-32 do not differ between SANC and LVC. In contrast, the reduced level of KEPI in SANC can be attributed to reduced KEPI transcripts, which are lower compared to those in LVC. Of note, in the context of reduced I-1 and DARPP-32 immunolabeling, PP1 protein immunolabeling in LVC exceeded that in SANC. It is important to note that other PP isoforms are expressed in SANC [35] but not assessed in the present study.

### 4.3. Phosphatase-Dependent Regulation of Ca^2+^ Clock Proteins

In the present study, PLB phosphorylation in SANC increased by about 2–3-fold in response to PP inhibition by CyA (Figure 3). An unexpected but rather interesting finding of our study was that following exposure to CyA, CaMKII-dependent phosphorylation of PLB at Thr^17^ increases rapidly, whereas at Ser^16^, the PKA-dependent site, the increase in phosphorylation occurs gradually (Figure 3). This result might indicate that PP activity within SANC is involved in temporal coordination of PKA and CaMKII-dependent phosphorylation.

Our results in permeabilized SANC indicate that PP inhibition by CyA induces a 20% increase in SR Ca^2+^ loading, resulting in a marked increase in the kinetics of Ca^2+^ cycling, as evidenced by a fourfold increase in total spontaneous Ca^2+^ release (the ensemble of LCR Ca^2+^ signal (Figure 4) per unit time). Thus, the effects on SR Ca^2+^ cycling suggest that PP inhibition not only increases Ca^2+^ pumping into SR but also accelerates the kinetics of the restitution phase between Ca^2+^ pumping and Ca^2+^ release and synchronization of the phases of spontaneous LCRs throughout the cell, reflecting increased availability of RyRs to become spontaneously activated. Rhythmicity of spontaneous diastolic LCRs in SANC appear to emerge from effects resulting from PKA and CaMKII-dependent protein phosphorylation, because when either PKA or CaMKII are inhibited, LCRs become small and irregular and generate a markedly suppressed ensemble Ca^2+^ signal, resembling Ca^2+^ sparks in VM [27], LCR occurrence is no longer rhythmic, but becomes stochastic. Indeed, following an increase in the SR Ca^2+^ cycling protein, phosphorylation in the presence of PDE and PP inhibition, rhythmic LCRs emerge in permeabilized LVC [16], confirming the key roles of PDE and PP in reducing cAMP phosphorylation in order to prevent the occurrence of rhythmic LCRs in LVC in the absence of Ca^2+^ overload.

Thus, protein phosphorylation synchronizes restitution mechanisms of spontaneous LCR occurrence in different loci throughout the cell, generating a rhythmic ensemble LCR Ca^2+^ signal of substantial amplitude, regardless of the cell type [16,27].

### 4.4. Phosphatase Regulation of Membrane Clock Proteins

In addition to the effects on SR Ca^2+^ cycling changes in the AP-triggered Ca^2+^ transient and AP firing rate in response to phosphatase inhibition also results, in part, from increased Ca^2+^ influx via L-type Ca^2+^ channels, as evidenced by an increase in the I_CaL_ amplitude and leftward shift of voltage dependence of I_CaL_ activation (Figure 8). This effect could result from an increase in the phosphorylation state of the channel subunits, as was shown in LVC, in which βAR stimulation increased the availability of L-type Ca^2+^ channels to activate in response to depolarization [36,37]. Recent evidence indicate that β-AR stimulation increases the availability of Ca_V_1.2 channels by enhancing its insertion from intracellular sites to the cell membrane [38].

Although PP2A binds directly to the C-terminal tail of Ca_V_1.2 [39] and PP1 regulates Cav1.2 channels in mouse VM [30,40], there is no proof that PP1 directly interacts with Ca_v_1.2 channels. The acceleration of AP repolarization in response to PP inhibition that accompanies an increase in the AP firing rate (Table S3) might be explained, in part at least, by increased phosphorylation of K^+^ channels due to signaling enabled by the AKAP (Yotiao) [41], enabling their increased availability to activate in response to membrane depolarization, as shown in LVC [42]. Recent studies also suggest that PKA also appears to phosphorylate HCN4 subunits to regulate funny current (I_f_), and that inhibition of PKA (which reduces phosphorylation, in addition to increasing cAMP), significantly impairs the β-AR stimulation-induced augmentation of I_f_ current in isolated murine SANC [43].

PPs can regulate pacemaker activity directly and indirectly via impacting other clock functions. We have previously demonstrated that even a small increase in [Na^+^]_i_ in response to Na^+^/K^+^ pump inhibition by the digitalis glycoside (digoxigenin) perturbs SANC automaticity via impacting numerous coupled-clock mechanisms by producing changes in Na^+^ and Ca^2+^ electrochemical gradients [44]. A subsequent numerical modeling study [45] validated these experimental results. Additionally, like the modulation of SERCA2A Ca^2+^ pumping by phosphorylation of PLB that modulates SERCA2A Ca^2+^ pumping, phosphorylation of phospholemman modulates Na^+^/K^+^ pump activity [46,47]. Of note, PP1 is a negative regulator of phospholemman phosphorylation [48]. In other terms, PP1 activation acts as a break on the regulation of the coupled-clock system mechanisms driven by Na^+^ and Ca^2+^ electrochemical gradients.

### 4.5. Numerical Model Simulations of Biophysical Effects of Phosphatase-Dependent Regulation of Coupled-Clock System in SANC

A numerical model of SANC, which configures pacemaker cell function as a coupled system of Ca^2+^ and M clocks [22], quantitatively predicated the biophysical effects of protein phosphatase inhibition observed in our experiments. Model simulations (Figure 9) predicted the experimentally observed increase in the AP firing rate and occurrence of earlier and stronger diastolic Ca^2+^ release in the context of increases in the SR Ca^2+^ pumping rate and I_CaL_, mimicking the experimental result of increased protein phosphorylation and increased I_CaL_ amplitude by CyA (Figure 3 and Figure 8). In other words, we included in the model the experimentally measured effects of phosphorylation to increase the SR Ca^2+^ pumping rate and I_CaL_ amplitude, and the model predicted the experimentally observed changes in AP firing rate as well as earlier and stronger diastolic LCR signal, a major feature of the coupled-clock mechanism, observed experimentally (Figure 7).

An important insight from our numerical modeling that cannot be obtained experimentally in AP firing SANC is that the biophysical mechanism of phosphatase-dependent regulation includes earlier and stronger activation of the respective diastolic inward I_NCX_, which, in turn, accelerates diastolic depolarization, ultimately accounting for the observed increase in spontaneous AP firing rate (Figure 9B).

Importantly, the observed AP firing rate increase is fully quantitatively explained only when protein phosphorylation of both clocks is increased, i.e., when the I_CaL_ increase is combined with SR Ca^2+^ pumping rate increase, and does not require changes in I_f_, either via shifts in the voltage of its half-maximum activation (i.e., cAMP-dependent mechanism) [33] or via an increase in I_f_ conductance [19].

That a major increase in RyR opening rate (k_s_), simulating possible effects of RyR phosphorylation has only a minor effect on the AP firing rate (Figure S3) is surprising and counterintuitive when taking into account that an increase in k_s_ actually decreases the AP firing rate. This apparent paradox can be explained on the basis of the coupled-clock theory: the AP rate is actually determined by the coupled-clock ticking, i.e., by the kinetics of SR refilling with Ca^2+^ but not by the RyR release per se. Our simulations show that if RyRs release Ca^2+^ prematurely (simulated via higher k_s_ in our model), the AP firing rate slows down. In other words, a stronger, premature Ca^2+^ release interferes with SR Ca^2+^ refilling [49] and slows clock ticking. This inhibition of SERCA2A Ca^2+^ pumping by an excessive amount of Ca^2+^ to be pumped may explain previous observations of a slower rate and arrhythmia caused: (i) by catecholaminergic polymorphic ventricular tachycardia (CPVT) in which RyR mutations result in excessive RyR release [50] and also (ii) by “leaky” RyR, producing excessive Ca^2+^ release in the presence of ryanodine [51].

Finally, it is important to note that the purpose of introducing numerical modeling into the present study was to demonstrate that phosphatase-dependent regulation of AP firing rate is executed via a coupled-clock mechanism. Other types of numerical modeling, e.g., improvements of the prototype model in Yaniv et al. [52], are required to link the complex biochemical reactions including the detailed phosphorylation/de-phosphorylation kinetics to the biophysical mechanisms and AP firing rate described in the present study.

## 5. Summary

In summary, the combined effects of PP inhibition are to increase basal phosphorylation of both SR proteins (PLB, RyR) and the surface membrane proteins (I_CaL_, I_K_) that modulate the basal state clock coupling and the spontaneous AP firing rate. The main functional effects of phosphorylation on both M and Ca^2+^ clock proteins is an increase in the size and an earlier occurrence of spontaneous RyR-generated LCRs (i.e., ensemble Ca^2+^ signal beneath the surface membrane) during diastolic depolarization, which shifts the acceleration of diastolic depolarization to earlier times, leading to a reduction in the AP cycle length [32]. The reduction in the LCR period, regulated by the entrainment of coupled-clock functions, predicts the reduction in AP cycle length (for a review see [1,53].Thus, basal PP activities reduce the extent to which clock molecules are phosphorylated in the basal state, suppressing the basal AP firing rate; basal PP activity, and basal PDE activity [11] act in concert to prevent excessive basal cAMP activation of PKA and CaMKII protein kinase activity, thereby limiting excessive basal phosphorylation of molecules that maintain cell Ca^2+^ homeostasis. The net result maintains the basal AP firing rate well below its maximum, enabling rapid, robust responses to external input aimed at affecting the AP firing rate. In other words, PP and PDE activities comprise a “double braking system” (Figure 1) on the feed-forward signaling of Ca^2+^-cAMP-PKA signaling that drives the SANC pacemaker function: PDEs are the “front brakes” and phosphatases are the “rear brakes” of the coupled-clock. In response to β-adrenergic stimulation, this “double breaking system” becomes partially overpowered, enabling cAMP and clock protein phosphorylation levels to increase. However, an increase in PKA-dependent phosphorylation further elevate PDE [54] and likely PP activities providing powerful negative feedback for cAMP signals and preventing excessive increases in cAMP and protein phosphorylation to limit the magnitude of the functional response to β -adrenergic receptor stimulation.

## Data Availability

The raw data supporting the conclusions of this article will be made available by the authors without undue reservation.

## Supplementary Materials

The following are available online at https://www.mdpi.com/article/10.3390/cells10113106/s1, Figure S1: Inhibition of PP1 and PP2A activity by CyA increases RyR phosphorylation at Ser2809 site, which is phosphorylated by both PKA and CaMKII. Figure S2: Representative confocal line-scan images of permeabilized SANC bathed in 150 nmol/L [Ca^2+^] in control conditions and following 2-min superfusion with 100 nmol/L okadaic acid (OKA). Figure S3: Results of the model sensitivity analysis examining possible involvement of RyR phosphorylation in regulation of SANC AP firing rate by phosphatases (PP). Table S1: PP’s and PP inhibitor’s alternative names of transcripts measured in RT-QPCR. Table S2: Primers and probes used for RT-QPCR. Table S3: AP parameters of isolated SANCs before and after inhibition of PP activity by different concentrations of CyA (* *p* < 0.05, Control vs CyA).

## Abbreviations

SAN: sinoatrial node; SANC, sinoatrial nodal cells; AC, adenylyl cyclase; AKAP, A-kinase-anchoring protein; AP, action potential; β-AR, beta adrenoceptors; CaMKII, Ca^2+^, calmodulin-dependent protein kinase II; CyA, calyculin A; DARPP-32, Dopamine- and cAMP-regulated phosphoprotein; I_f_, funny current; I_CaL_, L-type Ca^2+^ current; g_CaL_, I_CaL_ conductance; HCN, hyperpolarization-activated cyclic nucleotide-gated channel; I-1, protein phosphatase-1 Inhibitor 1; IBMX, isobutylmethylxanthine; KEPI, kinase C-enhanced PP1 inhibitor; LCR, local submembrane Ca^2+^ releases; LVC, left ventricular cardiomyocytes; NCX, Na^+^/Ca^2+^ exchanger; OKA, okadaic acid; PDE, phosphodiesterase; PKA, protein kinase A; PLB, phospholamban; PP, phosphoprotein phosphatase; PP1, protein phosphatase-1; PP2A, protein phosphatase-2A; RyR, ryanodine receptors; SR, sarcoplasmic reticulum.
