# Supplementary material for: Phosphoprotein Phosphatase 1 but Not 2A Activity Modulates Coupled-Clock Mechanisms to Impact on Intrinsic Automaticity of Sinoatrial Nodal Pacemaker Cells
Source: Cells. 2021 Nov 10;10(11):3106. doi: 10.3390/cells10113106 (PMC8623309; doi:10.3390/cells10113106)
Supplement: Supplementary file 1 [file cells-10-03106-s001.zip › cells-1409279-sp-conversion.pdf]

cells

MDPI

Supplementary Material

Isolation of cardiac cells

New Zealand White rabbits (Charles River Laboratories, Wilmington, MA) were treated in accordance with the NIH Guide for the Care and Use of Laboratory Animals. Sinoatrial nodal, and ventricular tissues were extracted from hearts of 2.5-3.3 kg animals, as previously described [1]. Spontaneously beating single sinoatrial node cells (SANC) were isolated from the rabbit heart according to the modified method of Ito and Ono [2] as previously described. Briefly, rabbits were deeply anesthetized with sodium pentobarbital (50–90 mg/kg). The heart was quickly removed and placed in the Tyrode solution containing (in mmol/L): NaCl, 130; $NaHCO_3$, 24; NaH_2PO_4, 1.2; $MgCl_2$, 1.0; $CaCl_2$, 1.8; KCl, 4.0; and glucose, 5.6; after continuous saturation with a mixture of 95% 0_2 and 5% $C0_2$ the pH was maintained at 7.4; temperature was maintained at 36° C. The sinoatrial node region was cut into small strips (~1.0 mm wide) perpendicular to the crista terminalis and excised and washed twice in Ca^{2+}-free Tyrode solution (34°C) containing (in mM): NaCl, 140; KCl, 5.4; $MgCl_2$, 0.5; NaH_2PO_4, 0.33; HEPES, 5; glucose, 5.5; pH= 6.9; then, incubated at 34°C for 30 min in Ca^{2+}- free Tyrode solution containing elastase type IV (0.6 mg/ml; Sigma, Chemical Co.), collagenase type 2 (0.6 mg/ml; Worthington, NJ, USA) and 0.1% bovine serum albumin (Sigma, Chemical Co.). Thereafter, the sinoatrial node preparation was washed in modified Kraftbruhe solution, containing: 70 mM potassium glutamate, 30 mM KCl, 10 mM KH_2PO_4, 1 mM $MgCl_2$, 20 mM taurine, 10 mM glucose, 0.3 mM EGTA, and 10 mM HEPES (titrated to pH 7.4 with KOH), and kept at 4°C for 1h in KB solution containing 50 mg/ml polyvinylpyrrolidone (PVP 40). Finally, cells were dispersed from the sinoatrial node preparation by gentle pipetting in the modified Kraftbruhe solution and stored at 4°C.

Adult left ventricular cardiomyocytes (LVC) were isolated from New Zealand White rabbit hearts [3]. After thoracotomy, hearts were rapidly excised and cannulated on a gravity driven Langendorff perfusion apparatus and perfused at 37 °C with a solution containing (in mM): NaCl 120, KCl 5.4, NaH_2PO_4 1.0, $NaHCO_3$ 20, glucose 10, $MgCl_2$ 1.6 (pH 7.2) bubbled with 95% O_2 and 5% CO_2. An initial wash of approximately 5 min was followed by perfusion with buffer containing 1 mg/ml collagenase (Worthington) and 0.02 mg/ml protease (type XIV, Sigma-Aldrich Corp). After 10-15 min the perfusion was stopped, and the hearts were minced with scissors and dissociated with transfer pipettes. The cells were centrifuged (100 × *g*) for 1 min and suspended into the above solution fortified with 250 μM $CaCl_2$. The cells placed for a second digestion for 10 to 15 min in a shaker (60 to 70 rpm) at 37°C, with Tyrode solution containing 100 mM $CaCl_2$ and collagenase (1 mg/ml). The cells were centrifuged (100 × *g*) for 1 min and suspended into the above solution fortified with 250 μM $CaCl_2$. $CaCl_2$ increased from 250 μM to 500 μM. Finally, after purification by gravity sedimentation, the cells were suspended in Tyrode buffer containing (in mM): NaCl 137, KCl 4.9, $MgCl_2$ 1.6, NaH_2PO_4 1.2, glucose 15, HEPES 20, and $CaCl_2$ 1 (pH 7.3 with NaOH). A subset of cells were washed and stored in Tyrode buffer containing no NaH_2PO_4.

RNA extraction, cDNA synthesis, RT- QPCR and data analysis

RNA was extracted from isolated rabbit left ventricular (LV), sinoatrial nodal cells (SANC) with RNeasy Mini Kit (Qiagen, Germantown, MD, USA) with DNAse on column digestion according to manufacturer protocol. 2 ug of total RNA was used for cDNA preparation in 50 ul reaction volume with MMLV reverse transcriptase (Life Technologies, Foster City, CA, USA) using manufacturer recommended conditions with random hexamers for priming. For each synthesis of cDNA we used no template control for the detection of possible contamination and no RT control for tracing of possible genomic DNA presence.

RT-QPCR was performed on ABI Prism 7900HT Sequence Detection System (Applied Biosystems, Foster City, CA, USA) with 384-well platform. Reaction was performed using TaqMan PCR Master Mix (Applied Biosystems, Foster City, CA, USA) or Platinum SybrGreen qPCR SuperMix-UDG (Invitrogen, Carlsbad, CA, USA) using manufacturer recommended conditions. For SybrGreen protocol the size of amplicon was verified and dissociation curve was obtained. Each well contained 0.5 ul of cDNA solution and 10 ul of reaction mixture. Each sample was quadruplicated and repeated twice using de novo synthesized cDNA sets. Preliminary reactions were performed for determination of efficiency of amplification. RT-QPCR analysis was performed using ddCt method. Expression levels of transcripts were normalized on expression of b-tubulin 2 (TUBB2A) level. For statistical analysis was used Student's *t*-test.

Primer selection

Only PPP1CA RNA rabbit sequence was available in GeneBank database. For the rest of the transcripts we used the following approach. First, we prepared alignments of human, mouse, and rat RNA sequences with identification of potential conserved regions. Second, most conserved regions were aligned against rabbit genomic DNA sequences (available at http://genome.ucsc.edu) and potential primer sites were selected. For each unconfirmed transcript we have selected 2 pair of primers for nested PCR in order to confirm specificity of selected fragments. When the sizes of amplicons were confirmed, we used verified regions of RNA sequences for selection of primers and probes (Table S2) with Primer Express 3.0 software (Applied Biosystems, Foster City, CA, USA) for following RT-QPCR using TaqMan or SybrGreen protocol (KEPI, DARPP-32).

Table S1. PP's and PP inhibitor's alternative names of transcripts measured in RT-QPCR.

Alias	GeneBank	Official Full Name
PP1	PPP1CA	Protein Phosphatase 1 Catalytic Subunit Alpha
PP2A	PPP2CA	Protein Phosphatase 2 Catalytic Subunit Alpha
I-1	PPP1R1A	Protein Phosphatase 1 Regulatory Inhibitor Subunit 1A
DARPP-32	PPP1R1B	Protein Phosphatase 1 Regulatory Inhibitor Subunit 1B
KEPI	PPP1R14C	Protein Phosphatase 1 Regulatory Inhibitor Subunit 14C
β-tubulin	Tubb2a	Tubulin Beta 2A Class IIa

Table S2. Primers and probes used for RT-QPCR .

Name		Sequence
PPP1CA	Fw	CAACCGCATCTACGGCTTCTAC
	Rv	GTTGAAGCAGTCGGTGAACGT
	Probe	ACGAGTGCAAGAGACGCTACAACATCAAGC
PPP2CA	Fw	CTGTGGTAACCAAGCTGCAATC
	Rv	GAGTAACATGTGGCTCGCCTCTA
	Probe	TCTTTCTTGCAGTTTGACCCAGCTCCTC
PPP1R1A	Fw	CCCCAGAGGTCGATGAAGAC
	Rv	CGTTGCCGAGGAGACATTG
	Probe	ATCCCCAACCCACTTCTCAAGCCCA
DARPP-32	Fw	TACACACCCCCCTCACTGAAA
	Rv	TCGCCCACGTTGCTGAT
KEPI	Fw	GAACTATGTTTGTGAATTTTCTG
	Rv	TAGCTTGTAAATAAATCATTGG
Tubb2a	Fw	CCAGTGCGGCAACCAGAT
	Rv	CGCTGTCGCCATGGTAACT

Western Blotting:

Basal abundance of endogenous PP and PPI

All chemicals and reagents were sourced from Sigma-Aldrich, Inc. (St. Louis, MO, USA) unless otherwise noted. For the detection of protein phosphatases and endogenous protein phosphatase inhibitors, cells isolated from rabbit left ventricle (LVC), sinoatrial node (SANC) were lysed in ice cold RIPA lysis buffer (25mM Tris•HCl pH 7.6, 150mM NaCl, 1% NP-40, 1% sodium deoxycholate, 0.1% SDS), 1mM PMSF and protease inhibitor cocktail (P8340; Sigma-Aldrich, Inc.) Insoluble cellular debris was removed from the samples by centrifugation at 10,000 x g and protein quantity of the supernatants determined by BCA assay (ThermoFisher Scientific, Waltham, MA, USA). 10-20 µg of cell lysate protein were resolved on 4-20% gradient TGX/SDS-PAGE gel (BioRad Laboratories, Hercules, CA, USA) and transferred to polyvinylidene difluoride (PVDF) membranes (BioRad Laboratories) using a BioRad TurboBlotter set at 1.3 Amp (25V) for 7 min. The membranes were blocked for 1 h at room temperature in 5% (wt/v) non-fat milk in Tris-buffered Saline (20 mM Tris and 150 mM NaCl, pH 7.6) with 0.1% Tween 20 (TBST). Membranes were then incubated overnight with antibody against PPP1CA (SAB5300221; Sigma-Aldrich, Inc., 1:2500), PPP2CA (06-222; Millipore, Bedford, MA, USA, 1:1000), Inhibitor 1 (ab40877; Abcam, Cambridge, MA, USA, 1:10000), KEPI (orb128558; Biorbyt, Cambridge, UK, 1:2000), DARPP-32 (sc-271111; Santa Cruz Biotechnology, Dallas, TX, USA, 1:1000) or Sarcomeric Alpha-Actinin (A7811; Sigma Aldrich, Inc., 1:5000) diluted in blocking buffer at 4°C followed by incubation with horse radish peroxidase conjugated secondary antibodies (Invitrogen, Waltham, MA, USA). Blots were developed with Pierce SuperSignal West Pico or West Dura ECL substrate kits (ThermoFisher Scientific) and protein band chemiluminescence captured on film (Phenix Research Products, Thomas Scientific, Swedesboro, NJ, USA) and quantified using Kodak MI SE software (Eastman Kodak, Rochester, NY, USA). Protein abundance values were normalized to sarcomeric alpha-actinin. Western data are displayed as mean ± S.E.M. (n=number of cell lysates).

Phospholamban phosphorylation

The detection of site specific PLB phosphorylation sites was performed in SANC as previously described [4]. Specifically, the SANC suspension was equally divided into 6 parts, and each part was individually treated: the first part with solvent control; the 2nd, 3rd, 4th, 5th, and 6th parts with PP1 and PP2 inhibitor, Calyculin A 100 nM for 1, 5, 10, 15 or 30 min respectively. The separate set of experiments has been performed to test dose-dependent effect of Calyculin A on Ser^{16} and Thr^{17} phosphorylation sites of PLB. SANC suspension was equally divided into 5 parts for phosphorylation at Ser16: the first part was treated with solvent control, the 2nd - 4th parts were treated with 1, 10, 50 and 1000 nM Calyculin A. For phosphorylation at Thr^{17} site SANC suspension was equally divided into 7 parts: the first part was treated with solvent control the 2nd - 7th parts were treated with 1, 5, 10, 50, 100 and 500 nM Calyculin A. All treatments were performed at 35±0.5°C. Cells were then solubilized to fully dissociate PLB into its monomeric form (6.7 KDa). Proteins were resolved by 7.5% urea/SDS-PAGE gel and transferred (10 µg protein/lane) to polyvinylidene difluoride (PVDF) membranes (Amersham Pharmacia Biotech). To detect PLB phosphorylation, antibodies specific either to P-Ser16 PLB phosphorylation site (1:10000, Badrilla) or to P-Thr17 PLB phosphorylation site (1:5000, Badrilla) and HRP conjugated secondary antibody (1:15000) (Bio Rad) were employed. PVDF membranes were exposed to chemiluminescence (ECL, Amersham Pharmacia Biotech) reaction and films were developed. Intensity of band was quantified by using ImageJ software (1.8V, Wayne Rasband, NIH).

Spontaneous APs and Ca^{2+} current recordings in intact sinoatrial nodal cells (SANC)

A perforated patch-clamp technique was employed to record action potentials (APs) and ruptured patch-clamp techniques was used to record currents, using Axopatch-200B patch-clamp amplifier (Axon Instruments, Foster City, CA) at 35±0.5°C [5,6]. Only regularly spontaneously beating spindle-shaped SANC were chosen for recordings of either APs or currents. The pipette solution for perforated patch studies contained (in mmol/L): K-gluconate, 120; NaCl, 5; MgATP, 5; HEPES, 5; KCl, 20; pH, 7.2. For perforated patch-clamp experiments, β-escin (25 µmol/L, Sigma) was added to the pipette solution [6]. The AP cycle length (CL) and AP characteristics were analyzed via a customized program [7,8]. The APCL was measured either as the interval between AP upstrokes (maximum dV/dt, V/s), or, in a subset of cells, as the interval between AP-induced Ca^{2+} transient. Other measured AP parameters also included: AP amplitude (AP amplitude); maximum diastolic potential (MDP); AP duration measured at 75% of repolarization time (APD_{75}); AP upstroke (dV/dt_{max}); diastolic depolarization rate (DD slope); threshold or take-off potential (TOP); time to non-linear diastolic depolarization (TNLDD) (from MDP to the onset of non-linear diastolic depolarization). We found that TNLLD highly correlated ($y = 1.0x + 90.7$, $R^2 = 0.7$, n=36 SANC) with AP ignition onset [7].

For recordings of $I_{Ca,L}$ (whole cell patch clamp), depolarizing voltage clamp pulses (300 ms) were applied from a holding potential of -50 mV; 10 µmol/L tetrodotoxin and 4 mmol/L 4-aminopyridine were added to block interfering currents. The bath solution contained the following (in mmol/L): NaCl, 117; TEA-Cl, 20; CsCl, 5.4; $MgCl_2$, 1; HEPES, 5; $CaCl_2$, 1.8; 4-AP, 4; pH = 7.4. The pipette solution contained the following (in mmol/L): NaCl, 10; TEA-Cl, 20; CsCl, 110; EGTA, 10; MgATP, 5; HEPES, 10; pH, 7.2. The bath temperature was maintained at 35±0.5°C.

Confocal imaging of AP-induced Ca^{2+} transient and local subsarcolemmal Ca^{2+} releases (LCR) in intact sinoatrial nodal cells (SANC)

Intact SANC were loaded with fluo-4 AM (Thermo Scientific, Waltham, MA, USA) (10µM for 15 min) (Molecular Probes, Inc., OR) and placed on the stage of Zeiss LSM-510 inverted confocal microscope (Carl Zeiss, Inc., Germany). Skinned SANC were loaded with 0.03 mM fluo-4 pentapotassium salt (Thermo Scientific, Waltham, MA, USA) and

placed on the stage of Zeiss LSM-410 inverted confocal microscope (Carl Zeiss, Inc., Germany). All images were recorded in the line-scan mode, with the scan line oriented along the long axis of the cell, close to sarcolemmal membrane and processed with IDL software (5.4, Research Systems, Boulder, CO).

The interval between the peaks of two adjacent AP-induced Ca^{2+}-transients is defined as AP-induced Ca^{2+}-transients cycle length. The LCR period is defined as the time from the peak of the prior AP-induced Ca^{2+} transient to an LCR peak in diastole. Other measurements included restitution of AP-induced Ca^{2+}transients $T_{50,90}$ (Ca^{2+}transients duration from the peak to 50, 90% of decay, ms).

The amplitude of each LCR was expressed as the peak value (F) normalized to minimal fluorescence (F_0), its spatial size was indexed as the full width at half maximum amplitude (FWHM), and its duration characterized as the full duration at half maximum amplitude (FDHM). The number of LCRs in permeabilized SANC was normalized per space and time interval (as previously described) [9].

SR Ca^{2+} content and local subsarcolemmal Ca^{2+} releases (LCRs) in permeabilized sinoatrial nodal cells (SANC)

A subset of cells was permeabilized with 0.01% saponin as previously described [10] in a solution containing in mmol/L: K aspartate, 100; KCl, 25; NaCl, 10; MgATP, 3; $MgCl_2$, 0.81 (free [Mg^{2+}] ~1 mM); HEPES, 20; EGTA, 0.5; phosphocreatine, 10 and creatine phosphokinase, 5U/ml; pH 7·2. The free [Ca^{2+}], at a given total Ca^{2+}, Mg^{2+}, ATP and EGTA concentration was calculated, using a computer program (WinMAXC 2.50, Stanford University, CA). After saponin washout, the solution was changed to the recording solution (same as above) that contained 0.03 mM fluo-4 pentapotassium salt and 150 nmol/L free $[Ca^{2+}]_i$. The bath temperature was maintained at 35±0.5°C. All images were recorded in the line-scan mode, with the scan line oriented along the long axis of the cell. Images were analyzed by custom-made imaging software and with IDL software (8.5, Research Systems, Boulder, CO). Power spectra was produced by a Clamp Fit program (version 9.2). The amplitude of individual LCRs was also expressed as a peak value (F) normalized to minimal fluorescence (F_0). LCR spatial size (FWHM) was indexed as the full width at half-maximum amplitude. LCR duration (FDHM) was characterized as the full duration at half-maximum amplitude. The number of LCRs was normalized per space and time interval.

Intracellular Ca^{2+} $[Ca^{2+}]_i$ in the cell was calculated from respective F/F_0 values using a formulation: $[Ca^{2+}]_i=K_d \cdot (F/F_0)/(K_d/[Ca^{2+}]_r+1-F/F_0)$, where $[Ca^{2+}]_r$ is $[Ca^{2+}]_i$ at rest, i.e. 150 nmol/L; K_d (1 μmol/L) is the dissociation constant for Ca^{2+}-bound fluo-4 . The Ca^{2+} released by each individual LCR was estimated as an individual LCR Ca^{2+} signal: FWHM×FDHM×1/2$\Delta F/F_0$ (where $\Delta F/F_0=F/F_0-1$ was recalculated to nmo/L $[Ca^{2+}]_i$ as previously described in [10], and the average individual LCR Ca^{2+} signal was then calculated for each cell. The spontaneous Ca^{2+} signal of the LCR ensemble was calculated as product of all individual LCRs normalized per space and time interval.

SR Ca^{2+} content in permeabilized SANC was assessed by application of caffeine (20 mM) by a 3 ms pressure pulse (Picospritzer II, General Valve Co) via a separate pipette placed closely (within ~100 μm) upstream to a tested cell. The average fluorescence of Fluo-4 signal (F/F_0) was calculated along the scan line, oriented perpendicular to the cell membrane, and reported as the amplitude of caffeine induced Ca^{2+} release, F/F_0 peak.

Immunolabeling of RyR2 in sinoatrial nodal cells (SANC)

Immunolabeling of RyR2 total and phosphorylated RyR2 at Ser^{2809} in SANC as previously described [11]. Freshly isolated rabbit SANC were incubated with normal Tyrode solution (control and negative control) or PP1 inhibitor CyA (Enzo Life Sciences (Farmingdale, NY, USA) (0.5 μM) at 37°C for 10 minutes. Then the cells were rinsed with PBS (no Ca^{2+} or Mg^{2+}), and fixed with 4% paraformaldehyde, permeablized with 1% Triton and

incubated with blocking solution (1×PBS containing 2% IgG-free BSA+ 5% donkey serum+0.02% NaN3+0.1% Triton) overnight. Cells were co-immunolabelled with antibody anti-total RyR2 (1:500, mouse, Sigma-Aldrich, St. Louis, MO, USA) and Atto 647N-conjugated anti-mouse IgG antibody (1:500, Sigma-Aldrich), and antibody anti-phosphorylated RyR2 at Ser^{2809} (1:200, rabbit, Badrilla, UK) and Cy3-conjugated anti-rabbit IgG antibody (1:1000, Jackson ImmunoResearch Laboratories). Dual confocal images of central sections of SANC were obtained with lasers 633 nm and 543 nm via a Zeiss LSM 510 (Carl Zeiss Inc., Germany). Only secondary antibodies were applied to the negative controls, which displayed negligible fluorescence. The average immunofluorescence density of a given cell (with the nuclear area excluded) was measured employing ImageJ software (1.8V, Wayne Rasband, National Institutes of Health). For semi-quantification, the phosphorylation level of a given cell was indexed by the average fluorescence density of phosphorylated RyR2 at Ser^{2809} normalized by the total RyR2 fluorescence density. Imaging presentation was obtained via LSM 5 Image Browser (Carl Zeiss Inc., Germany).

Numerical modeling

Numerical simulations employed a model of rabbit SANC that portrays the pacemaker cell function as a coupled system of Ca-clock and M-clock [12]. The model code is freely available at cellML website (http://models.cellml.org/workspace/maltsev_2009): the model can be downloaded and run in CellML format using the Cellular Open Resource software developed by Alan Garny at Oxford University in the UK (for recent development of this software see http://www.opencor.ws/). Quantitative effects of protein phosphorylation on the diastolic Ca^{2+} release characteristics and spontaneous beating rate were explored by increasing maximum SR Ca^{2+} pumping rate (P_{up}), mimicking effect of PLB phosphorylation at Ser^{16}, and simultaneously increasing maximum conductance of I_{CaL} (g_{CaL}) in accordance with our experimental results. The model integration was performed on a Xeon™ (3.2 GHz)-based workstation (Hewlett-Packard) with a fixed time step of 0.01 ms. The achievement of steady-state firing solution by the model was determined using the algorithm suggested previously by Kurata et al. [13]: numerical integration was continued until the relative differences in both amplitude and period between the newly calculated cycle and the preceding cycle became < 0.001.

Statistical Analysis

Data are displayed as mean ± S.E.M. Data were log-transformed when necessary to correct for unequal variances. Statistical significance of differences between means were tested by Student's *t*-test or analysis of variance (ANOVA), when appropriate. A value of $P<0.05$ is considered as statistically significant.

Drugs

Drugs were added to the bath solution before the experiment. Sodium fluoride, HEPES, protease inhibitor cocktail for mammalian cells, EGTA, DMSO, $MgCl_2$, $CaCl_2$ and okadaic acid (OKA) were from Sigma-Aldrich (St. Louis, MO, USA). PPs inhibitor, Calyculin A (CyA) was from Enzo Life Sciences (Farmingdale, NY, USA), BCA Protein assay kit was from Pierce. Antibodies were from Sigma-Aldrich (St. Louis, MO, USA). (PPP1CA, SAB5300221, Sarcomeric Alpha-Actinin, A7811), Millipore (PPP2CA, 06-222), Abcam (Inhibitor 1, ab40877), Biorbyt orb (KEPI, orb128558), Santa Cruz Biotechnology (DARPP-32, sc-271111), Badrilla (P-Ser16 PLB, P-Thr17 PLB, Total PLB), Invitrogen (radish peroxidase conjugated secondary antibodies), Bio Rad (HRP conjugated secondary antibody), Life Technologies Corporation (A11034). 96-Well Microplates were from Fisher scientific. Dithiothreitol was from Invitrogen; Triton® X-100 solution from Santa Cruz. ProFluor Ser/Thr PPase Assay was from Promega (V1260). Drugs for SANC permeabilization and Ca^{2+} cycling recording were purchased from Sigma-Aldrich (St. Louis, MO, USA) and PP1

and PP2A catalytic subunits were from New England BioLabs (Ipswich, MA, USA) and Promega (Madison, WI, USA).

ONLINE DATA SUPPLEMENT RESULTS.

Table S3. AP parameters of isolated SANCs before and after inhibition of PP activity by different concentrations of CyA (* $p < 0.05$, Control vs CyA).

AP parameters	Control ($n = 5$)	100 nM CyA ($n = 5$)	Control ($n = 5$)	300 nM CyA ($n = 5$)	Control ($n = 5$)	500 nM CyA ($n = 5$)
**SANC rate, beat/min**	173.4±8.3	197.6±12.0*	182.2±12.8	231.5±22.7*	187.4±9.2	246.5±9.8*
**SANC cycle, ms**	353.2±18.8	296.3±10.6*	340.1±23.7	274.4±30.6*	323.0±16.2	245.3±8.9*
**Amplitude, mV**	99.9±3.6	104.1±1.8	97.4±5.3	102.5±4.0	98.5±3.7	98.2±6.1
**MDP, mV**	-61.0±2.8	-62.2±1.0	-57.1±3.2	-62.3±3.0	-60.2±2.0	-59.2±2.2
**APD75, ms**	135.8±22.9	103.2±7.8	129.7±11.8	114.9±8.6	111.5±4.5	91.4±5.1*
**dV/dt max, V/sec**	13.9±2.7	17.0±2.2	13.2±2.8	16.8±3.1	17.4±1.5	20.8±0.7
**DD slope, mV/sec**	68.4±6.3	103.8±5.4*	75.8±10.5	105.8±17.7*	86.7±4.0	110.9±10.0*
**TOP, mV**	-48.3±2.7	-46.7±2.2	-46.0±1.9	-52.4±3.2	-45.3±2.2	-46.3±2.1
**TNLDD, ms**	167.8±10.2	121.1±9.8*	148.6±17.5	103.8±19.3*	172.17±21.2	119.58±10.7*

*TNLDD was highly correlated with AP Ignition [7].

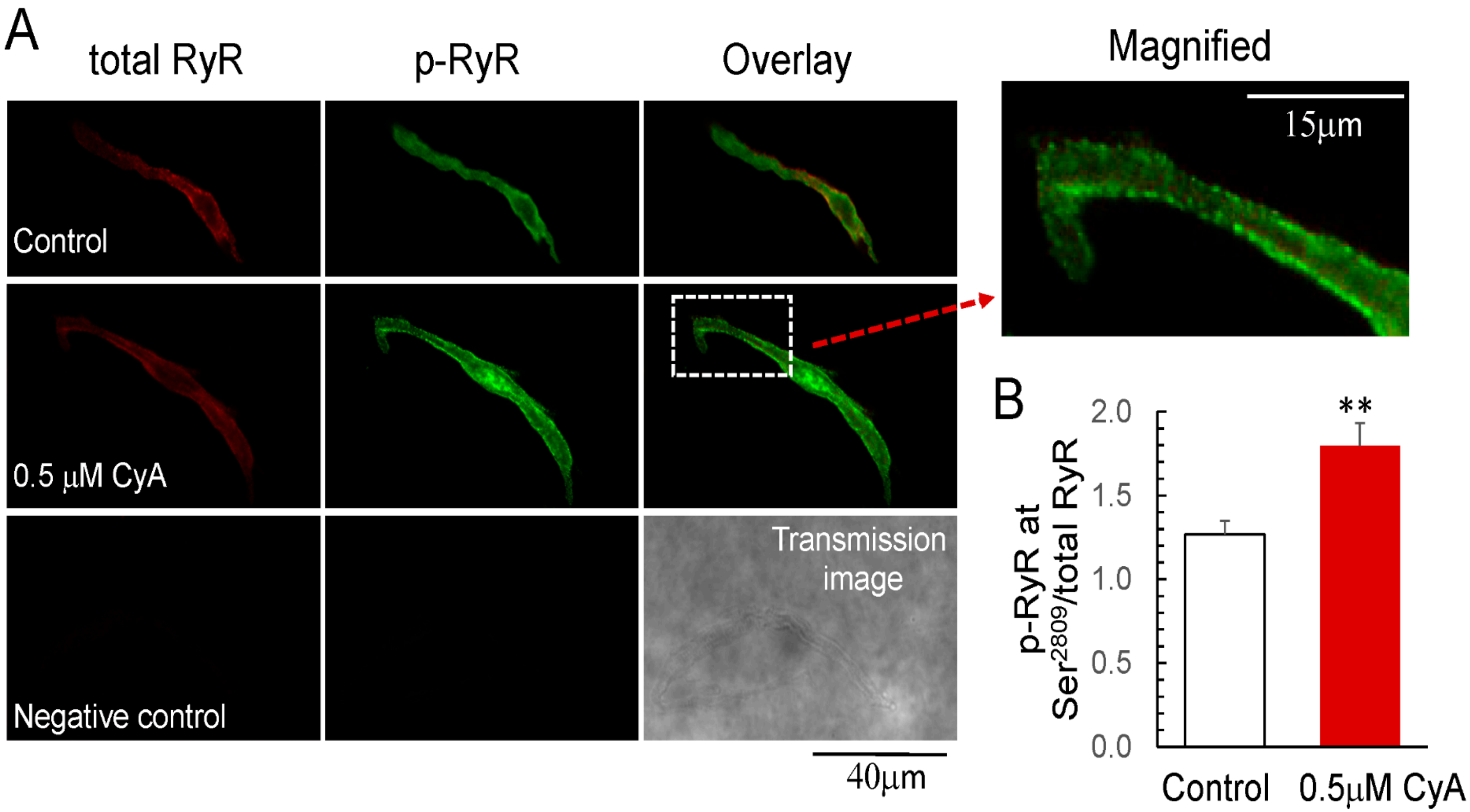

Figure S1. Inhibition of PP1 and PP2A activity by CyA increases RyR phosphorylation at Ser^{2809} site, which is phosphorylated by both PKA and CaMKII. (A) Representative confocal images of SANC immunolabeled for total RyR (red) and RyR phosphorylated at Ser^{2809} site (p-RyR, green) in control conditions and after treatment with CyA (magnified area of the cell is shown on the right). (B) Average changes in the phosphorylated-RyR at Ser^{2809} normalized to total RyR in SANC prior to and after treatment with CyA (n=68 or 56 cells for control or CyA, respectively). **P<0.01. Negative control shows staining with secondary antibodies and demonstrates negligible fluorescence.

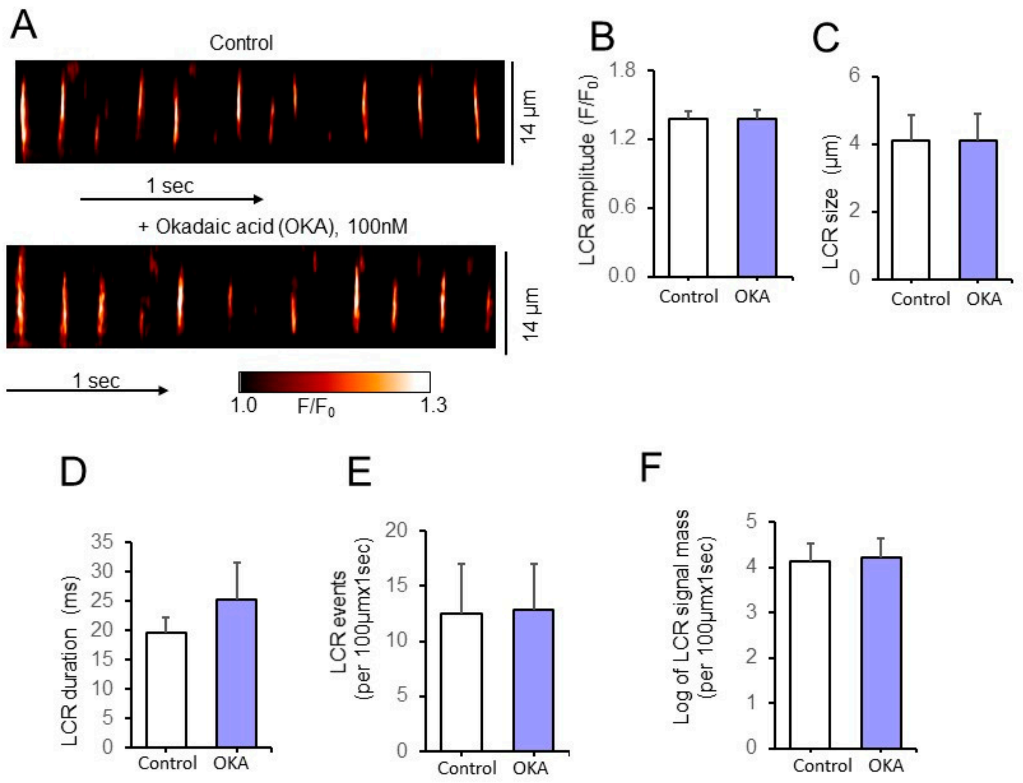

Figure S2. (A) Representative confocal line-scan images of permeabilized SANC bathed in 150 nmol/L [Ca^{2+}] in control conditions and following 2-minute superfusion with 100 nmol/L okadaic acid (OKA). (B-F) LCRs prior to and during treatment with OKA (n=5). (B) LCR amplitude (as normalized to Ca^{2+} fluorescence, F/F_0 peak); (C) LCR size (as FWHM, the full width at half-maximum amplitude); (D) LCR duration (as FDHM, the full duration at half-maximum amplitude); (E) Number of LCR events per 100 µm of the line-scan image and during a 1-sec time interval, (F) Ca^{2+} signals of the LCR ensemble (as the integrated Ca^{2+} signal of all LCRs). $P>0.05$, paired t-test.

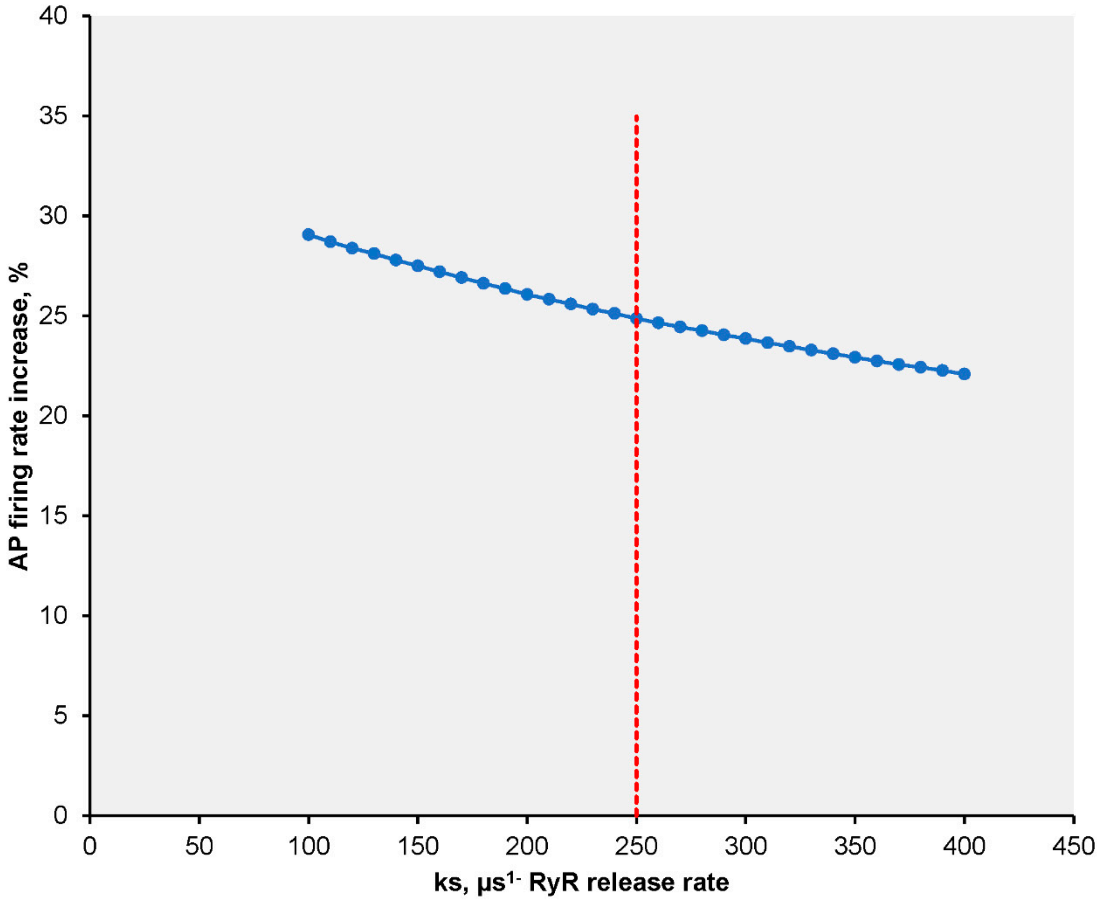

Figure S3. Results of the model sensitivity analysis examining possible involvement of RyR phosphorylation in regulation of SANC AP firing rate by phosphatases (PP). X axis: RyR opening rate was varied in a wide range from 100 μs^{-1} to 400 μs^{-1} (in steps of 10 μs^{-1}) that includes the value of 250 μs-1 of the basal state AP firing (shown by vertical line). These RyR changes were applied on the top of the respective g_{CaL} and Pup changes that simulate the 25% increase rate produced by PP inhibition by 100 nM of CyA in Main text Figure 9. Y axis: The combined effect of changes in RyR opening rate, g_{CaL}, and P_{up} to increase the AP firing rate.

References

1. Vinogradova, T.M.; Sirenko, S.; Lyashkov, A.E.; Younes, A.; Li, Y.; Zhu, W.; Yang, D.; Spurgeon, H.A.; Lakatta, E.G. Constitutive phosphodiesterase activity restricts spontaneous beating rate of cardiac pacemaker cells by suppressing local Ca^{2+} releases. *Circ. Res.* **2008**, *102*, 761–769.
2. Ito, H.; Ono, K. A rapidly activating delayed rectifier K+ channel in rabbit sinoatrial node cells. *Am. J. Physiol* **1995**, *269*, H443-452, doi:10.1152/ajpheart.1995.269.2.H443.
3. Lukyanenko, Y.O.; Younes, A.; Lyashkov, A.E.; Tarasov, K.V.; Riordon, D.R.; Lee, J.; Sirenko, S.G.; Kobrinsky, E.; Ziman, B.; Tarasova, Y.S.; et al. Ca2+/calmodulin-activated phosphodiesterase 1A is highly expressed in rabbit cardiac sinoatrial nodal cells and regulates pacemaker function. *J. Mol. Cell. Cardiol.* **2016**, *98*, 73–82, doi:10.1016/j.yjmcc.2016.06.064.
4. Kuschel, M.; Zhou, Y.Y.; Spurgeon, H.A.; Bartel, S.; Karczewski, P.; Zhang, S.J.; Krause, E.G.; Lakatta, E.G.; Xiao, R.P. beta(2)-adrenergic cAMP signaling is uncoupled from phosphorylation of cytoplasmic proteins in canine heart. *Circulation* **1999**, *99*, 2458–2465, doi:Doi 10.1161/01.Cir.99.18.2458.
5. Bogdanov, K.Y.; Vinogradova, T.M.; Lakatta, E.G. Sinoatrial nodal cell ryanodine receptor and Na(+)-Ca(2+) exchanger: Molecular partners in pacemaker regulation. *Circ. Res.* **2001**, *88*, 1254–1258, doi:10.1161/hh1201.092095.
6. Vinogradova, T.M.; Zhou, Y.Y.; Bogdanov, K.Y.; Yang, D.; Kuschel, M.; Cheng, H.; Xiao, R.P. Sinoatrial node pacemaker activity requires Ca^{2+}/calmodulin-dependent protein kinase II activation. *Circ. Res.* **2000**, *87*, 760–767.
7. Lyashkov, A.E.; Behar, J.; Lakatta, E.G.; Yaniv, Y.; Maltsev, V.A. Positive Feedback Mechanisms among Local Ca Releases, NCX, and ICaL Ignite Pacemaker Action Potentials. *Biophys J.* **2018**, *114*, 1176–1189, doi:10.1016/j.bpj.2017.12.043.
8. Lyashkov, A.E.; Juhaszova, M.; Dobrzynski, H.; Vinogradova, T.M.; Maltsev, V.A.; Juhasz, O.; Spurgeon, H.A.; Sollott, S.J.; Lakatta, E.G. Calcium cycling protein density and functional importance to automaticity of isolated sinoatrial nodal cells are independent of cell size. *Circ. Res.* **2007**, *100*, 1723–1731 doi:10.1161/CIRCRESAHA.107.153676.
9. Vinogradova, T.M.; Zhou, Y.Y.; Maltsev, V.; Lyashkov, A.; Stern, M.; Lakatta, E.G. Rhythmic ryanodine receptor Ca^{2+} releases during diastolic depolarization of sinoatrial pacemaker cells do not require membrane depolarization. *Circ. Res.* **2004**, *94*, 802–809.

10. Sirenko, S.; Yang, D.; Li, Y.; Lyashkov, A.E.; Lukyanenko, Y.O.; Lakatta, E.G.; Vinogradova, T.M. Ca(2)(+)-dependent phosphorylation of Ca(2)(+) cycling proteins generates robust rhythmic local Ca(2)(+) releases in cardiac pacemaker cells. *Sci Signal.* **2013**, *6*, ra6, doi:10.1126/scisignal.2003391.
11. Yang, D.; Lyashkov, A.E.; Li, Y.; Ziman, B.D.; Lakatta, E.G. RGS2 overexpression or G(i) inhibition rescues the impaired PKA signaling and slow AP firing of cultured adult rabbit pacemaker cells. *J. Mol. Cell. Cardiol.* **2012**, *53*, 687–694, doi:10.1016/j.yjmcc.2012.08.007.
12. Maltsev, V.A.; Lakatta, E.G. Synergism of coupled subsarcolemmal Ca2+ clocks and sarcolemmal voltage clocks confers robust and flexible pacemaker function in a novel pacemaker cell model. *Am. J. Physiol Heart Circ. Physiol* **2009**, *296*, H594-615, doi:10.1152/ajpheart.01118.2008.
13. Kurata, Y.; Hisatome, I.; Imanishi, S.; Shibamoto, T. Dynamical description of sinoatrial node pacemaking: Improved mathematical model for primary pacemaker cell. *Am. J. Physiol* **2002**, *283*, H2074–H2101.
